# Supplementary material for: Point-of-choice kilocalorie labelling practices in large, out-of-home food businesses: a preobservational versus post observational study of labelling practices following implementation of The Calorie Labelling (Out of Home Sector) (England) Regulations 2021
Source: BMJ Open. 2024 Apr 11;14(4):e080405. doi: 10.1136/bmjopen-2023-080405 (PMC11015320; doi:10.1136/bmjopen-2023-080405)
Supplement: Supplementary data [file bmjopen-2023-080405supp001.pdf]

Supplementary Materials

Table of contents

1. Section 1: List of Standard Industrial Classification codes used for sampling 2
2. Section 2: Full list of businesses assessed for kcal labelling practices 3
3. Section 3: Measures used to assess kcal labelling based on government guidance 4
4. Section 4: Power Analysis 5
5. Section 5: Supplementary Tables 6

Section 1: List of Standard Industrial Classification codes used for sampling

Large businesses with the following Standard Industrial Classification (SIC) codes (and hence in the following IDBR sections) that are subject to the policy and thus included in this study:

Section I (accommodation and food service activities)

Within section G:

- SIC 47.11- Retail sale in non-specialised stores with food, beverages or tobacco predominating.
- SIC 47.24- Retail sale of bread, cakes, flour confectionery and sugar confectionery in specialised stores.
- SIC 47.29 Other retail sales of food in specialised stores.

Within section R:

- SIC 91.03-Operation of historical sites and buildings and similar visitor attractions
- SIC 91.04-Botanical and zoological gardens and nature reserve
- SIC 93.11-Operation of sports facilities
- SIC 93.12-Activities of sports clubs
- SIC 93.13-Fitness facilities
- SIC 93.21-Activities of amusement parks and theme parks

Within section J:

- SIC 59.14-Motion picture projection activities

Section 2: Full list of businesses assessed for kcal labelling practices

ASK	Harris and Hoole	Radisson Blu
Beefeater Grill	Harvester	Reel Cinemas
Bella Italia	Hickory's Smokehouse	Revolution
Bespoke Hotels Ltd	Hilton Hotels & Resorts	S A Brain
Bills	Holiday Inn	Sainsbury's
Brasserie Blanc	Joe & The Juice	Shepherd Neame Pubs
Brewdog	John Lewis	Showcase Cinemas
Brewers Fayre	Kew Gardens	Sizzling Pub
Britannia Adelphi Hotel	KFC	Slug & Lettuce
Burger King	La Tasca	Soho Coffee Co.
Byron	Las iguana	Starbucks Coffee
Caffe Nero	Le Bistrot Pierre	Stone house restaurants
Campanile Hotels	Leon	Stonegate Pubs
Charles Wells Pub Company	M & S	Subway
Chiquito	Marriott	T G I Friday's
Cineworld	Marston’s	Tesco
Costa	McDonald's	The Ivy Cafe
Cote	Mercure	The Talbot Hotel
Crystal Leisure Ltd	Millennium & Copthorne Hotels	Tortilla
Dudley zoo	Miller & Carter	Travelodge
Ember Inns	Mitchells & Butler	Turtle Bay
Fayre-square	Morrisons Daily	Vintage Inns
Fish n chicken	Nando’s	Wagamama
Five Guys	Nicholson's Pubs	Walker Art Gallery
Franco Manca	Oak Tree Pubs	Wetherspoon
Frankie & Benny's	Odeon Cinemas	white 4brasserie
Fuller's	Pitcher and piano	Yee Rah
Gourmet Burger Kitchen	Pizza Express	YO SUSHI
Greene King	Pizza Hut	Young's
Hallmark Hotel Group	Premier Inn	Zizzi

Section 3: Measures used to assess kcal labelling practices based on government guidance (DHSC, 2018).

Kcal labelling Criteria	Rating	
1) Is kcal labelling provided at any point of choice? (e.g menus, display board)	Y	N
2) Is kcal labelling provided at all points of choice? (Menus, display boards etc.)	Y	N
3) Is kcal labelling provided for all eligible food items?	Y	N
4) Is kcal labelling provided for all non-alcoholic drink items?	Y	N
5) Is kcal labelling provided per portion for shareable items?	Y	N
6) Is kcal labelling presented close to the item's name or price?	Y	N
7) Is kcal labelling presented as prominently as name or price?	Y	N
8) Is kcal reference info for an adult woman displayed anywhere?	Y	N
9) Is kcal reference info displayed clearly and prominently?	Y	N

Section 4: Power Analysis

The sample size was determined based on 2018 data showing that 17% of large OHFO in the UK provided voluntary kcal labelling (Robinson et al, 2018). Based on a power calculation a minimum sample size of n=96 (24 outlets per local authority) at pre and post implementation would give 80% power to detect a doubling of prevalence from 17% to 34% at α=0.05, (assuming a pre-post correlation of 0.1). We therefore aimed to recruit a minimum of 96 outlets at pre and post implementation.

Supplemental material
BMJ Publishing Group Limited (BMJ) disclaims all liability and responsibility arising from any reliance placed on this supplemental material which has been supplied by the author(s)
BMJ Open

Section 5: Supplementary Tables

Table 1: Effects of labelling regulations (pre-post), index of multiple deprivation, local authority and outlet type on presentation of any kcal labelling in unique outlets.

	**Any kcal labelling**		
Exposure variables	*Odds Ratios*	*99% CI*	*p*
Time (post-regulations)	25.64	3.72 – 176.87	**<0.001**
IMD-2	0.41	0.06 – 2.92	0.241
IMD-3	1.23	0.20 – 7.41	0.771
IMD-4	0.15	0.01 – 2.87	0.097
IMD-5	0.32	0.03 – 3.31	0.210
LA Liverpool	0.83	0.14 – 5.01	0.790
LA Milton Keynes	0.83	0.14 – 4.93	0.787
LA Richmond	1.42	0.15 – 13.84	0.690
Outlet Type (entertainment, retail, attraction)	0.62	0.10 – 3.87	0.498
Outlet Type (restaurants)	0.48	0.12 – 1.89	0.169
**Random Effects**			
σ^2	3.29		
$\tau_{00\ outletid}$	1.08		
ICC	0.25		
$N_{outletid}$	90		
Marginal R^2 / Conditional R^2	0.410 / 0.566		

Legend: IMD = Index of multiple deprivation; LA = Local authority. Reference categories were IMD = 1; Local authority of Dudley; cafes, fast-food, pubs and hotels for outlet type.

Table 2: Effects of labelling regulations, index of multiple deprivation, local authority and outlet type on total compliance score for kcal labelling for unique outlets.

	**Total Compliance Score (0-9)**		
Exposure variables	*Incidence Rate Ratios*	*99% CI*	*p*
Time (post-regulations)	5.19	3.83 – 7.04	**<0.001**
IMD-2	0.79	0.38 – 1.65	0.408
IMD-3	1.11	0.57 – 2.16	0.687
IMD-4	0.50	0.16 – 1.52	0.108
IMD-5	0.74	0.32 – 1.71	0.351
LA Liverpool	0.83	0.42 – 1.62	0.464
LA Milton Keynes	0.86	0.45 – 1.67	0.567
LA Richmond	1.06	0.45 – 2.47	0.862
Outlet Type (entertainment, retail, attraction)	0.83	0.42 – 1.66	0.494
Outlet Type (restaurants)	0.89	0.54 – 1.45	0.527
**Random Effects**			
σ^2	0.37		
$\tau_{00\ outletid}$	0.45		
ICC	0.55		
$N_{outletid}$	90		
Marginal R^2 / Conditional R^2	0.470 / 0.760		

Legend: IMD = Index of multiple deprivation; LA = Local authority. Reference categories were IMD = 1; Local authority of Dudley; cafes, fast-food, pubs and hotels for outlet type.

Table 3: Interactions between time and index of multiple deprivation for compliance total score.

	Compliance Total Score (0-9)		
Exposure variables	*Incidence Rate Ratios*	*99 % CI*	*p*
Time (post-regulations)	3.68	2.52 – 5.38	**<0.001**
IMD-2	0.05	0.00 – 0.67	**0.003**
IMD-3	1.35	0.69 – 2.63	0.244
IMD-4	0.22	0.04 – 1.09	0.015
IMD-5	0.30	0.13 – 0.69	**<0.001**
Time * IMD-2	18.73	1.42 – 246.54	**0.003**
Time * IMD-3	0.74	0.41 – 1.37	0.211
Time * IMD-4	3.44	0.72 – 16.44	0.042
Time * IMD-5	2.97	1.33 – 6.62	**<0.001**
**Random Effects**			
σ^2	0.33		
$\tau_{00\ outletid}$	0.31		
ICC	0.49		
$N_{outletid}$	114		
Observations	228		
Marginal R^2 / Conditional R^2	0.713 / 0.853		

Legend: IMD = Index of multiple deprivation; LA = Local authority. Reference categories were IMD = 1.

Table 4: Interactions between time and local authority for compliance total score.

	**Compliance Total Score (0-9)**		
Exposure variables	*Incidence Rate Ratios*	*99% CI*	*p*
Time (post-regulations)	6.25	3.58 – 10.91	**<0.001**
LA Liverpool	1.34	0.61 – 2.98	0.342
LA Milton Keynes	1.28	0.58 – 2.81	0.427
LA Richmond	0.80	0.33 – 1.89	0.496
Time * LA Liverpool	0.69	0.33 – 1.43	0.189
Time * LA Milton Keynes	0.65	0.31 – 1.36	0.135
Time * LA Richmond	1.05	0.47 – 2.37	0.874
**Random Effects**			
σ^2	0.33		
$\tau_{00\ outletid}$	0.35		
ICC	0.51		
$N_{outletid}$	114		
Observations	228		
Marginal R^2 / Conditional R^2	0.511 / 0.762		

Legend: LA = Local authority. Reference categories were local authority of Dudley.

Table 5: Interactions between time and outlet type for compliance total score.

	Compliance Total Score (0-9)		
Exposure variables	*Incidence Rate Ratios*	*CI*	*p*
Time (post-regulations)	3.52	2.57 – 4.82	**<0.001**
Outlet Type (entertainment, retail, attraction)	1.22	0.58 – 2.57	0.501
Outlet Type (restaurants)	0.11	0.04 – 0.34	**<0.001**
Time * Outlet Type (entertainment, retail, attraction)	0.68	0.35 – 1.35	0.148
Time * Outlet Type (restaurants)	10.66	3.58 – 31.78	**<0.001**
**Random Effects**			
σ^2	0.33		
$\tau_{00\ \text{outletid}}$	0.35		
ICC	0.52		
N_{outletid}	114		
Observations	228		
Marginal R^2 / Conditional R^2	0.711 / 0.860		

Legend: Reference categories were cafes, fast-food, pubs and hotels for outlet type.
