# Supplementary material for: Point-of-choice kilocalorie labelling practices in large, out-of-home food businesses: a preobservational versus post observational study of labelling practices following implementation of The Calorie Labelling (Out of Home Sector) (England) Regulations 2021
Source: BMJ Open. 2024 Apr 11;14(4):e080405. doi: 10.1136/bmjopen-2023-080405 (PMC11015320; doi:10.1136/bmjopen-2023-080405)
Supplement: Author's manuscript [file bmjopen-2023-080405.draft_revisions.pdf]

BMJ Open

BMJ Open is committed to open peer review. As part of this commitment we make the peer review history of every article we publish publicly available.

When an article is published we post the peer reviewers' comments and the authors' responses online. We also post the versions of the paper that were used during peer review. These are the versions that the peer review comments apply to.

The versions of the paper that follow are the versions that were submitted during the peer review process. They are not the versions of record or the final published versions. They should not be cited or distributed as the published version of this manuscript.

BMJ Open is an open access journal and the full, final, typeset and author-corrected version of record of the manuscript is available on our site with no access controls, subscription charges or pay-per-view fees (http://bmjopen.bmj.com).

If you have any questions on BMJ Open's open peer review process please email info.bmjopen@bmj.com

BMJ Open

Point-of-choice kilocalorie labelling practices in large, out-of-home food businesses before and after the implementation of kilocalorie Labelling (Out of Home Sector) (England) Regulations 2021: An Observational Study

Journal:	*BMJ Open*
Manuscript ID	bmjopen-2023-080405
Article Type:	Original research
Date Submitted by the Author:	29-Sep-2023

Complete List of Authors:	Polden, Megan; University of Liverpool, Department of Primary Care and Mental Health; Lancaster University<br>Jones, Andrew; Liverpool John Moores University<br>Essman, Michael; University of Cambridge, MRC Epidemiology Unit<br>Adams, Jean; University of Cambridge, MRC Epidemiology Unit<br>Bishop, Tom; University of Cambridge, MRC Epidemiology Unit<br>Burgoine, Thomas; University of Cambridge, MRC Epidemiology Unit<br>Donohue, Aisling; Liverpool John Moores University<br>Sharp, Stephen; University of Cambridge, MRC Epidemiology Unit<br>White, Martin; University of Cambridge, MRC Epidemiology Unit<br>Smith, Richard ; University of Exeter Medical School, Institute of Health Research<br>Robinson, Eric; University of Liverpool, Department of Psychology

Keywords:	Nutrition < TROPICAL MEDICINE, Public health < INFECTIOUS DISEASES, Health policy < HEALTH SERVICES ADMINISTRATION & MANAGEMENT, Obesity

SCHOLARONE™ Manuscripts

I, the Submitting Author has the right to grant and does grant on behalf of all authors of the Work (as defined in the below author licence), an exclusive licence and/or a non-exclusive licence for contributions from authors who are: i) UK Crown employees; ii) where BMJ has agreed a CC-BY licence shall apply, and/or iii) in accordance with the terms applicable for US Federal Government officers or employees acting as part of their official duties; on a worldwide, perpetual, irrevocable, royalty-free basis to BMJ Publishing Group Ltd ("BMJ") its licensees and where the relevant Journal is co-owned by BMJ to the co-owners of the Journal, to publish the Work in this journal and any other BMJ products and to exploit all rights, as set out in our licence.

The Submitting Author accepts and understands that any supply made under these terms is made by BMJ to the Submitting Author unless you are acting as an employee on behalf of your employer or a postgraduate student of an affiliated institution which is paying any applicable article publishing charge ("APC") for Open Access articles. Where the Submitting Author wishes to make the Work available on an Open Access basis (and intends to pay the relevant APC), the terms of reuse of such Open Access shall be governed by a Creative Commons licence – details of these licences and which Creative Commons licence will apply to this Work are set out in our licence referred to above.

Other than as permitted in any relevant BMJ Author's Self Archiving Policies, I confirm this Work has not been accepted for publication elsewhere, is not being considered for publication elsewhere and does not duplicate material already published. I confirm all authors consent to publication of this Work and authorise the granting of this licence.

For peer review only

Title: Point-of-choice kilocalorie labelling practices in large, out-of-home food businesses before and after the implementation of kilocalorie Labelling (Out of Home Sector) (England) Regulations 2021: An Observational Study

For peer review only

Word count: 4012

Author names and affiliations:

Megan Polden, Department of Primary Care and Mental Health, University of Liverpool

Andrew Jones, Aisling Donohue, Liverpool John Moores University

Michael Essman, Jean Adams, Tom Bishop, Thomas Burgoine, Stephen J. Sharp, Martin White, MRC Epidemiology Unit, University of Cambridge

Richard Smith, Faculty of Health and Life Sciences, University of Exeter

Eric Robinson, Department of Psychology, University of Liverpool

Corresponding author information: Megan Polden, Department of Primary Care and Mental Health, University of Liverpool, Liverpool, UK, m.polden@liverpool.ac.uk

Abstract

Background and Objectives: On 6th April 2022 the UK government implemented mandatory kilocalorie (kcal) labelling regulations for food and drink products sold in the out-of-home food sector (OHFS) in England. Previous assessments of kcal labelling practices in the UK OHFS found a low prevalence of voluntary implementation and poor compliance with labelling recommendations. This study aimed to examine changes in labelling practices pre vs. post-implementation of mandatory labelling regulations in 2022.

Methods: In August-December 2021 (pre-implementation) and August-November 2022 (post-implementation), large OHFS businesses (250 or more employees) subject to labelling regulations were visited. At two time points a researcher visited the same 117 food outlets (belonging to 90 unique businesses) across four local authorities in England. Outlets were rated for compliance with government regulations for whether kcal labelling was: provided at any or all point-of-choice, provided for all eligible food and drink items, provided per portion for sharing items, if labelling was clear and legible and if kcal reference information was displayed.

Results: There was a significant increase (21% pre-implementation vs. 80% post-implementation, OR = 40.98 [95% CI: 8.08 to 207.74], $p<0.001$) in the proportion of outlets providing any kcal labelling at point-of-choice post-implementation. Only 15% of outlets met all labelling compliance criteria post-implementation, with a minority of outlets not presenting labelling in a clear (33%) or legible (29%) way.

Conclusion: The number of large businesses in the OHFS providing kcal labelling increased following the implementation of mandatory labelling regulations. However, around one-fifth of eligible outlets sampled were not providing kcal labelling 4-8 months after the regulations came into force, and the majority of businesses only partially complied with government

For peer review only

guidance. More effective enforcement may be required to further improve kcal labelling practices in the OHFS in England.

Registration: Study protocol and analysis strategy pre-registered on Open Science Framework (https://osf.io/pfnm6/).

Keywords: Kcal labelling, nutrition, public health, out-of-home food

For peer review only

Article Summary

Strengths and Limitations:

- This is the first study in England to investigate kcal labelling compliance in the out-of-home food sector pre vs. post-implementation of mandatory kcal labelling regulations.
- This study had high inter-rater reliability when examining adherence to labelling guidance and assessed a range of OHFS businesses from multiple local authorities sampled from across the socio-economic spectrum.
- The study only examined large OHFS businesses and therefore findings are not reflective of the whole OHFS
- The current study did not examine consistency across different outlets within a chain and therefore conclusions cannot be drawn on the level of consistency of kcal labelling within large chains.

Introduction

The prevalence of obesity has been steadily rising both globally and in the UK since 1980[1,2], with 64% of adults and 38% of 10–11-year-olds in England classified as having overweight or obesity[3]. Between 25% and 39% of UK adults reported eating food from the out-of-home food sector (OHFS) at least once per week in 2008-12 and 2018[4-6] . Food purchased from the OHFS is often high in energy[7,8] and frequent consumption of this food is associated with poorer diet quality and overweight/obesity[4,9,10]. A 2021 study in England found that on average people purchased 1013 kcals per meal when eating out of the home and consumed an average of 915 kcals[11], significantly higher than the UK government recommendation of 600 kcals or less for a lunch or evening meal[12]. Consumers also tend to underestimate the energy content of food purchased from the OHFS[13,14]. In addition, poor-quality diet and obesity are associated with a lower socioeconomic position (SEP)[15] and people of lower SEP are more likely to report being less motivated by weight management or healthiness of food choices[16], which may contribute to SEP-related differences in OHFS behaviour.

Multiple countries and regions, including the US[17] and Canada[18], have introduced mandatory policies that require OHFS outlets to provide consumers with point-of-choice kcal labelling for food and drink products. Meta-analytic evidence from randomised control trials and longitudinal studies indicates that kcal labelling may have small public health benefits, as the provision of kcal labelling has been shown to reduce the number of kcals purchased in food outlets[19]. However, other reviews have concluded that kcal labelling has a negligible effect on consumer behaviour[20]. Effects are likely mediated to some extent by the quality of labelling implemented and these labels being noticed and used. Thus, high-quality implementation will likely be important to maximise the effects of kcal labelling on consumer purchasing and consumption.

For peer review only

In 2011, the UK government launched the public health responsibility deal with companies making voluntary pledges to improve public health – including providing kcal labelling in the OHFS and best practice guidelines on how to provide labelling[21]. The extent to which this pledge resulted in business practice change was unclear, as evaluations were reliant on self-reported and incomplete data from businesses[22]. In a 2018 study, only 18 out of 104 (17%) unique business OHFS outlets in England provided in-store kcal labelling[23]. Furthermore, the quality of this kcal labelling was inconsistent, often lacking prominence and clarity, and not presented on all available food and drink items[23]. The overall quality of labelling presented is likely to impact whether customers notice and use kcal labelling when selecting products[24].

Motivated by the lack of voluntary kcal labelling compliance in the OHFS, the UK government consulted on mandating kcal labelling in 2018. Following public consultation[25], legislation was passed in July 2021, with mandatory kcal labelling in large food businesses required as of 6th April 2022[26,27]. The regulations require large (250 or more employees) businesses (cafes, fast-food outlets, sit-down restaurants, pubs) in England selling food and non-alcoholic drinks in scope of the regulations to: (1) display the energy content of food in kcals; (2) reference the size of the portion to which the kcal information relates; and (3) display the statement that 'adults need around 2000 kcal a day'. Food and non-alcoholic drinks are in scope of the regulations if they are: (1) offered for sale in a form which is suitable for immediate consumption; (2) not prepacked food; and (3) not exempt food such as food on a temporary menu for less than 30 consecutive days[28].

To date, only one study has examined OHFS adherence to mandatory kcal labelling legislation[29]. This US study found an overall high level of compliance (94%) across 197 chain restaurants subject to federal menu labelling law (20 or more US sites). Similar data is not currently available following the implementation of regulations in England and evidence

For peer review only

on how OHFS kcal labelling practices changed following the implementation of mandatory kcal labelling will be able to inform any future development of the regulations.

This study aimed to compare kcal labelling practices in large OHFS businesses in England subject to kcal labelling regulations before (2021) and after (2022) mandatory labelling regulations in 2022. We examined changes in the proportion of outlets providing kcal labelling and the level of compliance with government guidelines regarding the clarity, prominence and legibility of the labelling; overall and by type of outlet and local authority deprivation.

Methods

Area and Outlet Sampling

Sampling of outlets was conducted in four purposively selected local government areas (i.e. local authorities (LA)) that ensured geographical coverage and representation across quintiles of deprivation (Index of Multiple Deprivation (IMD)[30] used at the local authority level). Local authority average IMD levels (1-5) were used with IMD1 reflecting the most deprived areas and IMD5 reflecting the least deprived. The four LAs sampled were Liverpool (IMD1 northern region), Dudley (IMD2 midlands), Milton Keynes (IMD3 South) and Richmond upon Thames (IMD5 London).

The Inter-Department Business Register[31] was used to identify eligible businesses likely to be subject to the mandatory kcal labelling policy within local authorities of interest (data sampled in June 2021, list produced in Autumn 2020). This is a list of all UK businesses, which includes their core characteristics, number of employees and principal activities defined using the Standard Industrial Classification. We identified Standard Industrial Classification codes likely to include businesses serving food (for the full list of Standard Industrial Classification codes we used see supplementary materials) and then identified large businesses

For peer review only

with 250 or more employees. Individual businesses often have multiple outlets (e.g. chain restaurants), so individual outlets belonging to each identified large business[32] within the four local authorities of interest were identified using Ordnance Survey Points of Interest[33] data from September 2020.

The sample size was determined based on 2018 data showing that 17% of large OHFOs in the UK provided voluntary kcal labelling[23]. Based on a power calculation (see online supplementary materials for full details), to detect a doubling of prevalence from 17% to 34%, we estimated we required a minimum sample size of 96 outlets. We used stratified sampling by LA and business type to sample a total of 117 outlets, representing 90 unique businesses, due to the limited availability of unique outlets in some local authorities. We prioritised sampling from unique business outlets (as opposed to sampling multiple outlets from the same business) as we assumed kcal practises would be more likely to differ between, as opposed to within, the same large businesses in the OHFS.

Three outlets closed in the post-policy period and were therefore excluded from the final analysis, resulting in a sample size of n=114 (87 unique businesses). This sample included outlets from attractions, cafes, fast food, hotels, pubs, restaurants, retail, and entertainment businesses (full list of businesses in supplementary materials) across the four local authorities.

Procedure

The kcal labelling assessment procedure was based on previous methods used to assess kcal labelling practises of large OHFS businesses in England during 2018[23]. Researchers visited selected outlets in August-November 2021 (pre-policy) and August-November 2022 (post-policy) to examine if kcal labelling was present and whether it adhered to best practice recommendations provided by the Department of Health and Social Care. In each outlet, researchers examined point-of-choice menu display boards inside and outside the outlet,

For peer review only

handheld food and drink menus and menus presented at ordering points. A researcher rated the presence of any kcal labelling (0=no, 1=yes), as well whether kcal labelling adhered to the following criteria: (1) provided at any point-of-choice (2) provided at all points-of-choice, (3) provided for all eligible food items, (4) provided per portion for sharing items, (5) presented close to the item's name and price, (6) presented as prominently as name or price, (7) provided alongside any kcal reference information, (8) provided alongside clearly and prominently displayed kcal reference information, (9) provided for all non-alcoholic drink items (See Table 1). A score between 0-9 was calculated for each outlet based on the number of kcal labelling practices the outlet adhered to. For example, a score of 3 would mean that the outlet adhered to 3 of the best practice recommendations (list of criteria in Table 1 and supplementary materials). For the post-implementation assessment, three additional kcal labelling criteria relating to whether labelling was clearly presented, legible and whether a kcal-free menu was available on request were added for further descriptive information (Table 1) based on kcal labelling guidance introduced alongside the new legislation[34].

Researchers received training on assessing outlet adherence to best practice recommendations for kcal labelling and the study protocol before the start of data collection to ensure consistency between raters. Ten per cent (n=17) of outlets were independently coded by a second researcher with a percentage agreement score of 96% across all 9 variables for the pre-assessment and 97% across all 12 variables for the post-assessment.

Data Analysis

A descriptive overview is presented for kcal labelling practices pre and post-implementation by outlet type and IMD quintile of the postcode of the location at the lower layer super output area[35] (LSOA) level. Due to the small number of outlets for some outlet types (e.g. Entertainment and Attractions), we combined categories into three meaningful

For peer review only

categories with adequate numbers: Restaurants; Entertainment, Retail and Attractions; Cafes, Fast-Food, Pubs, and Hotels.

To examine whether kcal labelling practices changed from before to after implementation and whether outlet characteristics were associated with kcal labelling regulations and compliance, regression models (logistic and Poisson) were used. Exposure variables were time (pre vs. post-implementation), outlet type (entertainment and retail venues; cafes, fast-food and pubs; and restaurants), deprivation level (LSOA IMD 1-5 (categorical)) and local authority (Liverpool, Dudley, Milton Keynes, Richmond Upon Thames). Where the outcome variable was kcal labelling implemented in any form (yes/no), a logistic regression model was used. Where the outcome variable was the total compliance score (0-9), a Poisson regression model was used. Unadjusted models included only the time variable. Adjusted models additionally included outlet type, deprivation level (IMD of LSOA) and local authority. We repeated these analyses limiting them to unique outlets. In instances where data was available from multiple outlets within a business, compliance data was used from the first outlet in which data was collected.

In subsequent pre-registered exploratory models, we planned to examine whether changes in compliance following labelling regulations differed by outlet type, local authority and deprivation level. However, some of these three-way interaction analyses were not possible due to small sample sizes (Ns < 5, e.g. only 2 restaurants had kcal information at baseline and only 4 entertainment/retail/attraction outlets did not report kcal information at post) meaning these interaction analyses would have been severely underpowered[36]. However, we conducted simple interaction analyses between time and IMD (at both IMD of LSOA and neighbourhood of outlet level), and time and outlet type for total compliance scores.

Finally, for the subset of outlets that had kcal labelling present at the pre-assessment, we performed the analysis described above (excluding interactions) to examine whether

For peer review only - http://bmjopen.bmj.com/site/about/guidelines.xhtml

labelling regulations were associated with improvements in labelling quality among outlets already providing kcal labelling. This approach supplements the main analyses, by addressing improvements in labelling quality among existing compliant outlets, as changes in overall labelling quality in the primary analyses may be driven by outlets introducing labelling (as opposed to also increasing compliance with guidelines for how labelling is provided).

To account for the relatively large number of analyses conducted, confidence intervals and p-values were set to 99% and < .01 respectively to determine statistical significance. R and R Studio version 1.2.503 was used to conduct analyses, using the 'lme4', 'lmertest', 'sjPlot', 'descr' and 'performance' packages. Code and data are available here [https://osf.io/gp3rf/]. The study did not involve human participants and made use of publicly available information, therefore ethical approval was not required. The study protocol and analysis strategy were pre-registered on Open Science Framework (https://osf.io/pfnm6/), and the data/analysis code is available here (https://osf.io/cg4wz/).

For peer review only

Results

Table 1 shows adherence to the policy pre-and post-implementation of the regulations.

Table 1. Level of compliance with kcal labelling guidelines in outlets pre-and post-implementation.

	Pre- and post-implementation assessments in all outlets[1] (n=117)		Pre- and post-implementation assessments limited to outlets with labelling present pre and post (n=24)	
	Pre-implementation assessment N (%)	Post-implementation assessment N (%)	Pre-implementation assessment N (%)	Post-implementation assessment N (%)
Kcal labelling provided at any point of choice	24 (21%)	91 (80%)	24 (100%)	24 (100%)
Kcal labelling provided at all points of choice	16 (14%)	76 (67%)	16 (67%)	22 (92%)
Kcal labelling provided for all food items	17 (15%)	80 (76%)	17 (70%)	16 (67%)
Kcal labelling provided per portion for sharing menu items	10 (9%)	19 (17%)	10 (42%)	7 (29%)
Kcal labelling presented close to the item's name and price	21 (18%)	91 (80%)	21 (88%)	22 (92%)
Kcal labelling presented as prominently[1] as name or price	0 (0%)	7 (6%)	0 (0%)	2 (8%)
Kcal reference information displayed anywhere	11 (9%)	83 (73%)	11 (45%)	21 (88%)
Kcal reference information displayed clearly and prominently	4 (3%)	51 (45%)	4 (17%)	12 (50%)
Kcal labelling provided for all non-alcoholic drink items	11 (9%)	79 (69%)	11 (45%)	21 (88%)
**Additional data only available for post-implementation assessment**				
Kcal labelling legible[2]		76 (67%)		19 (79%)
Kcal labelling presented clearly[3]		81 (71%)		19 (79%)
Non-kcal menu available on request		11 (12%)		0 (0%)

[1]Note: Pre-implementation total outlets = 117 individual outlets from across 90 unique businesses. Post-implementation total outlets= 114 individual outlets from across 87 unique businesses post-implementation.

[1] The prominence of the labelling was determined based on whether the labelling was of equal prominence as item name or price i.e. text size and colour

[2] The legibility was determined by whether the kcal labelling was clear enough to read i.e. font size, font type and colour of text

[3] The clarity of the labelling was determined based on the kcal labelling was easy to understandable i.e. multiple kcal options presented close together or multiple kcal options requiring the customer to do additional sums to determine the kcal content.

For peer review only

Outcome: Kcal labelling presented in any form.

An unadjusted model with time (pre-implementation vs. post-implementation) demonstrated a significant effect with OR = 40.98 [95% CI: 8.08 to 207.74], Z = 4.48, $p < .001$: Marginal $R^2 = 0.40$), indicating an increased odds of kcal labelling presented in any form post-implementation of about 41 times than seen pre-implementation.

In an adjusted model (Table 2) the effect of time remained significant. However, the IMD quintile of outlet location, outlet type and local authority were not significantly associated with any kcal labelling.

Table 2: Effects of labelling regulations, index of multiple deprivation, local authority and outlet type on presentation of any kcal labelling

	**Any kcal labelling**		
Exposure variables	*Odds Ratios*	*99% CI*	*p*
Time (post-implementation)	35.11	4.87 – 252.98	**<0.001**
IMD-2	0.24	0.03 – 1.76	0.065
IMD-3	1.17	0.23 – 5.86	0.804
IMD-4	0.17	0.02 – 1.88	0.058
IMD-5	0.21	0.02 – 1.89	0.067
LA Liverpool	1.43	0.27 – 7.65	0.580
LA Milton Keynes	0.76	0.15 – 3.79	0.658
LA Richmond	1.63	0.19 – 13.67	0.555
Outlet Type (entertainment, retail, attractions)	0.50	0.09 – 2.89	0.307
Outlet Type (restaurants)	0.49	0.15 – 1.65	0.130
**Random Effects**			
σ^2	3.29		
$\tau_{00\ outletid}$	1.07		
ICC	0.24		

For peer review only

$N_{outletid}$	114
Marginal R^2 / Conditional R^2	0.465 / 0.596

Legend: IMD = Index of multiple deprivation; LA = Local authority. Reference categories were IMD = 1; Local authority of Dudley; cafes, fast-food, pubs and hotels for outlet type.

There was some evidence of multi-collinearity between LA (VIF = 3.57) and IMD quintile of LSOA (VIF = 4.50). Removal of either variable did not affect the models. We repeated the analyses limiting them to unique outlets. The overall pattern of results was similar; post-implementation led to OR = 25.64 (95% CI: 3.72 – 176.87) increased odds for any kcal labelling, in the full model (see online supplementary materials).

Overall compliance score

A multilevel Poisson model with a random intercept of outlet examined overall compliance score (0 – 9). An unadjusted model with time (pre-implementation vs. post-implementation) demonstrated a significant effect with IRR = 5.06 [95% CI: 3.90 to 6.56], Z = 16.10, $p < .001$: Marginal $R^2 = 0.49$), indicating that compliance score was 6 times greater post vs pre-implementation of the regulations. Pre-implementation, the mean compliance score was 1.00/9 (2.18) and post-implementation it was = 5.06/9 (2.80). In an adjusted model (Table 3) time remained significant and the IMD quintile at the LSOA level, outlet type and local authority were not significant predictors of the overall compliance score.

Table 3: Effects of labelling regulations, index of multiple deprivation, local authority and outlet type on total compliance score for kcal labelling

	**Total Compliance Score (0-9)**		
Exposure variables	*Incidence Rate Ratios*	*99% CI*	*p*
Time (post-implementation)	5.06	3.90 – 6.56	**<0.001**
IMD-2	0.70	0.38 – 1.28	0.128
IMD-3	1.10	0.67 – 1.80	0.623
IMD-4	0.61	0.29 – 1.29	0.088
IMD-5	0.66	0.34 – 1.29	0.110
LA Liverpool	0.99	0.59 – 1.65	0.948
LA Milton Keynes	0.85	0.51 – 1.40	0.395
LA Richmond	1.10	0.56 – 2.17	0.704
Outlet Type (entertainment, retail, attraction)	0.82	0.48 – 1.43	0.366
Outlet Type (restaurants)	0.94	0.65 – 1.36	0.650
**Random Effects**			
σ^2	0.33		
$\tau_{00\ outletid}$	0.30		
ICC	0.48		
$N_{outletid}$	114		
Marginal R^2 / Conditional R^2	0.528 / 0.754		

Legend: IMD = Index of multiple deprivation; LA = Local authority. Reference categories were IMD = 1; Local authority of Dudley; cafes, fast-food, pubs and hotels for outlet type.

There was some evidence of multi-collinearity between local authority (VIF = 3.50) and IMD quintile (VIF = 3.50). Removal of either variable from the model did not

For peer review only

substantially influence the results. We repeated the analyses limiting them to only unique outlets. The overall pattern of results was similar; post-implementation led to IRR = 5.19 (95% CI: 3.83 – 7.04) increase in any kcal labelling, in the full model (see online supplementary materials).

In separate models, we examined the interaction between time and IMD, time and LA, and time and outlet type. There were no significant interactions between time and LA (ps > .135). There was a significant interaction between time and outlet type, specifically restaurants (IRR = 10.66 [95% CI: 3.58 to 31.78], p < .001). Compared to cafès, fast-food, pubs and hotels, there were greater increases in compliance for kcal labelling in restaurants post-implementation (increase in compliance scores in restaurants = 5.33, increase in cafes, fast-food, pubs and hotels = 3.54). Finally, there was a significant interaction between time and IMD, specifically for IMD2 (IRR = 18.73 [95% CI: 1.42 – 246.54], p = .003) and IMD5 (IRR = 2.97 [95% CI: 1.33 to 6.62], p < .001). There was a larger increase in compliance scores for IMD2 (increase = 4.86) and IMD5 (increase = 4.34), compared to IMD1 (increase = 3.92). Full model reporting can be found in online supplementary tables 3-5.

Exploratory analysis: Examining changes in compliance score among 24 outlets that displayed kcal at both time periods

We limited our analysis to the 24 outlets which had any kcal labelling at pre-implementation and examined whether labelling regulations increased the total compliance score. There was no evidence that the total compliance scores significantly increased from pre to post-implementation in these outlets (IRR = 1.25 [95% CI: 0.97 – 1.59], z = 1.75, p = .081), indicating that the change in compliance score observed in the primary analysis was largely driven by more outlets providing any form of kcal labelling, as opposed to compliance to recommendations significantly increasing in outlets already providing kcal

For peer review only

labelling. At the pre-implementation time point mean compliance score = 4.75/9 (2.17) and at post mean compliance score = 5.92/9 (2.28). Frequency data on individual labelling criteria pre vs. post for the subset of 24 outlets is presented in Table 1.

Discussion

Summary of main findings

This is the first study we are aware of to examine point-of-choice kcal labelling practices in the OHFS before and after the implementation of mandatory kcal labelling regulations in England. Overall, results indicated that labelling regulations were associated with a significant increase in the likelihood of OHFS outlets providing kcal labelling, with 21% in 2021 compared to 80% in 2022 providing labelling in any form. Compliance with government guidelines for nine kcal labelling best practice criteria also increased post-implementation. The changes indicate the policy had a significant impact on labelling practices and prevalence in the OHFS. However, it should be noted that although compliance overall increased, there was still a substantial number of eligible outlets not providing any form of kcal labelling post-implementation of the policy (20%). In addition, there remained a lack of compliance with labelling guidelines, particularly in relation to presenting labelling and kcal reference information clearly and prominently; only 17/114 outlets (15%) met all guideline criteria post-implementation. When analyses were limited to outlets already implementing kcal labelling pre-legislation, there was no evidence of a change in compliance scores for labelling best practice criteria. This finding suggests that the increase in overall compliance scores from pre to post-implementation observed in the full sample was strongly driven by outlets introducing new labelling, as opposed to substantial improvements in the overall quality of existing labelling.

For peer review only

Twenty per cent of eligible outlets sampled were not providing any in-store kcal labelling at the time of post-implementation assessment. This reflects a rate of non-compliance greater than that observed in a US study, where only 6% of eligible outlets were not implementing kcal labelling in a similar number of unique businesses[29]. Of the outlets that provided in-store kcal labelling in our study, most were not presented as prominently as name or price, presented at all points of choice, presented on all eligible food and drink items and for some outlets, it was not presented clearly or legibly. These results are consistent with research conducted prior to the announcement of the 2022 kcal labelling policy which found that existing voluntary kcal labelling did not align with government recommendations in the UK OHFS[23]. We also examined the legibility and clarity of kcal labelling post-regulations as this was specified in the legislation. The majority of outlets sampled post-regulations presented kcal labelling that was legible (67%) and clear (71%), but it often lacked prominence (94% of kcal labelling rated as not as prominent as name or price). The level of impact of mandatory kcal labelling on consumer behaviour may be dependent on consumers' level of engagement[37] and this may be mediated by the prevalence and prominence of kcal labelling in the OHFS. Whether kcal labelling is implemented and labelling is clearly and prominently presented is likely to impact whether customers notice and use kcal labelling when selecting products[24].

There was a significant interaction between pre-post implementation and outlet type, specifically with restaurants showing a greater increase in compliance compared to cafes, fast-food, pubs and hotels. This interaction may have been driven by the particularly low compliance levels of restaurants at the pre-implementation time point compared to other outlet types. Furthermore, there was a significant interaction between time and IMD, specifically that outlets from IMD quintiles 2 and 5 showed a statistically significant larger increase in compliance scores compared to IMD1. This may indicate that outlets located in

For peer review only

more affluent areas showed greater improvement in kcal labelling practice post-regulations. However, the sample sizes in these analyses are small and further research examining the robustness of these associations is needed. This difference may also have been driven by the prevalence of different outlet types in each area, for example proportionally more fast-food establishments (as opposed to pubs) in Liverpool compared to Richmond Upon Thames.

Providing kcal information at the point of choice in the OHFS is hypothesised to support consumers in making informed dietary decisions and some, but not all, evidence indicates that this leads to consumers purchasing lower kcal and healthier food options[38]. As a proportion of major OHFS businesses are not complying with the kcal labelling regulations this may limit the impact on consumer behaviour.

Recent qualitative data suggested that local authorities are not engaging in pro-active enforcement, and instead relying on the presumed compliance of large businesses who they expect to comply with regulations to protect their reputations and maintain customer trust[39]. However, our study has found that 20% of eligible outlets are not adhering to kcal labelling regulations in any form and 85% are not complying with labelling guidance in full. Our findings suggest that greater enforcement by local authorities may be required to improve current labelling practices in the UK OHFS.

There has been some public concern about the implementation of mandatory kcal labelling in England[37], specifically about the potential negative impacts on people with eating disorders[40]. In an attempt to mitigate potential negative impacts, as part of government guidance for kcal labelling, it was recommended that kcal-free menus be available in outlets on request. Our results found that only 12% of outlets implementing kcal labelling were able to provide researchers with a kcal-free menu when requested in-store. Therefore, attempts to mitigate the potential harms of kcal labelling on vulnerable groups may require more formal

For peer review only

guidance or a mandate to make the practice of kcal-free menus available on request more widespread.

Strengths and limitations

To our knowledge, this is the first study that has assessed and compared kcal labelling practices in the OHFS before and after the implementation of mandatory kcal labelling regulations in England. Data collection for kcal labelling assessments had high inter-rater reliability between researchers. This study assessed a range of OHFS businesses from multiple local authorities sampled from across the socio-economic spectrum in England, however, it is not clear how representative this sample is. As this study focused on large businesses subject to the kcal labelling policy, we did not examine kcal labelling practices in outlets exempt from the policy, including smaller businesses (<250 employees) and independent outlets, so this study does not give a full representation of kcal labelling practices across all businesses in the OHFS. Furthermore, it should be noted that exploratory analyses examining local area and outlet type variability in pre-post implementation changes to labelling were based on small sample sizes.

Additionally, this study examined labelling practices at a single time point post-implementation (approximately 4-8 months post-implementation) and it could be argued that some outlets needed more time to implement the regulations and comply with guidance. However, the government consulted on mandating kcal labelling in 2018 with a public consultation occurring in 2020. The legislation was then passed in 2021 with labelling being required from April 2022. This timeline of events provided businesses with ample time to prepare and enact required regulations. Therefore, it is unlikely that businesses did not have sufficient time to implement labelling regulations ahead of this labelling assessment and it is unlikely this is a legitimate reason for a lack of or poor compliance in some outlets in this assessment.

For peer review only

Future Research

Our study examined large businesses in the OHFS subject to mandatory kcal labelling regulations. There has been debate as to whether to expand these regulations to include medium and small OHFS businesses and future research could examine whether kcal labelling regulations also motivated smaller businesses not subject to the policy to provide voluntary kcal labelling on menus. Additionally, we were unable to examine whether labelling is provided consistently within outlets of the same business and this could be explored in future research. Research is also yet to examine the nutritional accuracy of newly implemented kcal labelling in the OHFS in England and future research would benefit from examining this.

Conclusions

Overall, the findings demonstrate a significant increase in kcal labelling practices in the OHFS in England following the implementation of the labelling regulations. However, there was still a substantial proportion of outlets not complying with the kcal labelling post-implementation, not meeting best-practice guidance and providing kcal labelling in a way that undermines the likelihood of it being used by consumers. More proactive enforcement may be required to further improve kcal labelling practice in the UK OHFS and businesses should be encouraged to review their labelling practices to ensure continued compliance with labelling regulation guidelines.

Funding statement: This report is independent research commissioned and funded by the Department of Health and Social Care Policy Research Programme (Implementation and assessment of mandatory kcal labelling in the out-of-home sector, NIHR200689). The views expressed in this publication are those of the author(s) and not necessarily those of the NIHR or the Department of Health and Social Care. JA, TB, TB, ME, SS and MW are supported by the MRC Epidemiology Unit, University of Cambridge [UKRI grant number MC/UU/00006/7]. MP receives support from the NIHR Applied Research Collaboration

For peer review only

ARC NWC and Alzheimer's Society and is funded through a Post-Doctoral Fellowship. ER is funded by the National Institute for Health and Care Research (NIHR) Oxford Health Biomedical Research Centre (BRC), Economic and Social Research Council (ES/W007932/1) and European Research Council (Grant reference: PIDS, 803194). The views expressed are those of the authors and not necessarily those of the funders, NHS or Department of Health and Social Care. For the purpose of Open Access, the author has applied a Creative Commons Attribution (CC BY) licence to any Author Accepted Manuscript version arising.

Competing interests statement: The authors declare no conflict of interest.

Author Contributions: Funding was obtained by J.A, E.R, A.J, M.W, R.S, T.B and S.S. Conceptualisation and methodology design contributions and project administration contributions were made by all authors. The formal analysis, data curation, and original draft preparation contributions were made by M.P, E.R and A.J. All authors contributed to revising the manuscript. All authors read and agreed to the published version of the manuscript.

Availability of data and materials: Data from this study is available on the Open Science Framework (https://osf.io/cg4wz/).

References

1. Ng M, Fleming T, Robinson M, Thomson B, Graetz N, Margono C, Mullany EC, Biryukov S, Abbafati C, Abera SF, Abraham JP. Global, regional, and national prevalence of overweight and obesity in children and adults during 1980–2013: a systematic analysis for the Global Burden of Disease Study 2013. The lancet. 2014 Aug 30;384(9945):766-81.
2. Boutari C, Mantzoros CS. A 2022 update on the epidemiology of obesity and a call to action: as its twin COVID-19 pandemic appears to be receding, the obesity and dysmetabolism pandemic continues to rage on. Metabolism. 2022 Aug 1;133:155217.
3. Office for National Statistics. Obesity Profile: short statistical commentary. July 2022. https://www.gov.uk/government/statistics/obesity-profile-update-july-2022/obesity-profile-short-statistical-commentary-july-2022#further-information
4. Lachat C, Nago E, Verstraeten R, Roberfroid D, Van Camp J, Kolsteren P. Eating out of home and its association with dietary intake: a systematic review of the evidence. Obesity reviews. 2012 Apr;13(4):329-46.

For peer review only

5. Adams J, Goffe L, Brown T, Lake AA, Summerbell C, White M, Wrieden W, Adamson AJ. Frequency and socio-demographic correlates of eating meals out and take-away meals at home: cross-sectional analysis of the UK national diet and nutrition survey, waves 1–4 (2008–12). International Journal of Behavioral Nutrition and Physical Activity. 2015 Dec;12(1):1-9.
6. Mills S, Adams J, Wrieden W, White M, Brown H. Sociodemographic characteristics and frequency of consuming home-cooked meals and meals from out-of-home sources: cross-sectional analysis of a population-based cohort study. Public health nutrition. 2018 Aug;21(12):2255-66.
7. Roberts SB, Das SK, Suen VM, Pihlajamäki J, Kuriyan R, Steiner-Asiedu M, Taetzsch A, Anderson AK, Silver RE, Barger K, Krauss A. Measured energy content of frequently purchased restaurant meals: multi-country cross sectional study. bmj. 2018 Dec 12;363.
8. Robinson E, Jones A, Whitelock V, Mead BR, Haynes A. (Over) eating out at major UK restaurant chains: observational study of energy content of main meals. bmj. 2018 Dec 12;363.
9. Rosenheck R. Fast food consumption and increased caloric intake: a systematic review of a trajectory towards weight gain and obesity risk. Obesity reviews. 2008 Nov;9(6):535-47.
10. Taher AK, Evans N, Evans CE. The cross-sectional relationships between consumption of takeaway food, eating meals outside the home and diet quality in British adolescents. Public health nutrition. 2019 Jan;22(1):63-73.
11. Polden M, Jones A, Adams J, Bishop T, Burgoine T, Essman M, Sharp SJ, Smith R, White M, Robinson E. Kilocalorie labelling in the out-of-home sector: an observational study of business practices and consumer behaviour prior to implementation of the mandatory calorie labelling policy in England, 2022. BMC Public Health. 2023 Dec;23(1):1-2.
12. Public Health England. Calorie reduction: the scope and ambition. 2018. https://www.gov.uk/government/publications/calorie-reduction-the-scope-and-ambition-for-action.
13. Elbel B. Consumer estimation of recommended and actual calories at fast food restaurants. Obesity. 2011 Oct;19(10):1971-8.
14. Block JP, Condon SK, Kleinman K, Mullen J, Linakis S, Rifas-Shiman S, Gillman MW. Consumers’ estimation of calorie content at fast food restaurants: cross sectional observational study. BMJ. 2013 May 23;346:f2907.
15. Adams J. Addressing socioeconomic inequalities in obesity: Democratising access to resources for achieving and maintaining a healthy weight. PLoS Medicine. 2020 Jul 28;17(7):e1003243.
16. Robinson E, Jones A, Marty L. The role of health-based food choice motives in explaining the relationship between lower socioeconomic position and higher BMI in UK and US adults. Int J Obes. 2022;21:1–7.
17. Cleveland LP, Simon D, Block JP. Compliance in 2017 with Federal Calorie Labeling in 90 chain restaurants and 10 retail food outlets prior to required implementation. American journal of public health. 2018 Aug;108(8):1099-102.
18. Goodman S, Vanderlee L, White CM, Hammond D. A quasi-experimental study of a mandatory calorie-labelling policy in restaurants: Impact on use of nutrition

information among youth and young adults in Canada. Preventive medicine. 2018 Nov 1;116:166-72.
19. Crockett RA, King SE, Marteau TM, Prevost AT, Bignardi G, Roberts NW, Stubbs B, Hollands GJ, Jebb SA. Nutritional labelling for healthier food or non-alcoholic drink purchasing and consumption. Cochrane Database of Systematic Reviews. 2018(2).
20. Robinson E, Marty L, Jones A, White M, Smith R, Adams J. Will calorie labels for food and drink served outside the home improve public health?. Bmj. 2021 Jan 20;372.
21. Jebb SA. Calorie labelling on the high street. BMJ. 2011 Jul 26;343.
22. Knai C, Petticrew M, Durand MA, Eastmure E, James L, Mehrotra A, Scott C, Mays N. Has a public–private partnership resulted in action on healthier diets in England? An analysis of the Public Health Responsibility Deal food pledges. Food policy. 2015 Jul 1;54:1-0.
23. Robinson, E., Burton, S., Gough, T., Jones, A. and Haynes, A., 2019. Point of choice kilocalorie labelling in the UK eating out of home sector: a descriptive study of major chains. *BMC Public Health*, *19*, pp.1-6.
24. Nikolaou CK, Hankey CR, Lean ME. Calorie-labelling: does it impact on calorie purchase in catering outlets and the views of young adults?. International Journal of Obesity. 2015 Mar;39(3):542-5.
25. Department of Health & Social Care. Mandating calorie labelling in the out-of-home sector. Government response to public consultation. Mandating calorie labelling in the out-of-home sector: government response to public consultation (publishing.service.gov.uk).2020
26. Kaur A, Briggs A, Adams J, Rayner M. New calorie labelling regulations in England. bmj. 2022 May 4;377.
27. Department of Health & Social Care. Mandating calorie labelling in the out-of-home sector. 2020. https://assets.publishing.service.gov.uk/government/uploads/system/uploads/attachment_data/file/903714/Calorie_Labelling_-_Consultation_Response.pdf
28. Department of Health & Social Care. The Calorie Labelling (Out of Home Sector) (England) Regulations 2021. 2021. The Calorie Labelling (Out of Home Sector) (England) Regulations 2021 (legislation.gov.uk)
29. Cleveland LP, Simon D, Block JP. Federal calorie labelling compliance at US chain restaurants. Obesity Science & Practice. 2020 Apr;6(2):207-14.
30. National Statistics. English indices of deprivation. 2019. https://www.gov.uk/government/statistics/english-indices-of-deprivation-2019
31. Evans P, Welpton R. 'Business Structure Database: The Inter-Departmental Business Register (IDBR) for Research'. Economic & Labour Market Review. 2009;3(6):71-5.
32. Hughes JC, James G, Evans A, Prestwood D. Implementation of Standard Industrial Classification 2007: December 2009 update. Economic & Labour Market Review. 2009 Dec 1;3:51-5.
33. Points of interest: technical information. https://beta.ordnancesurvey.co.uk/products/points-of-interest
34. Department of Health & Social Care. The Calorie Labelling (Out of Home Sector) (England) Regulations 2021. 2021. https://www.legislation.gov.uk/uksi/2021/909/regulation/6/made

35. Office for National Statistics. Rural urban classification (2011) of lower layer super output areas in England and Wales. 2011.
36. Leon AC, Heo M. Sample sizes required to detect interactions between two binary fixed-effects in a mixed-effects linear regression model. Computational statistics & data analysis. 2009 Jan 15;53(3):603-8.
37. Polden M, Robinson E, Jones A. Assessing public perception and awareness of UK mandatory calorie labelling in the out-of-home sector: Using Twitter and Google trends data. Obesity Science & Practice. 2022 Jul 11.
38. Petimar J, Zhang F, Rimm EB, Simon D, Cleveland LP, Gortmaker SL, Bleich SN, Polacsek M, Roberto CA, Block JP. Changes in the calorie and nutrient content of purchased fast food meals after calorie menu labeling: A natural experiment. PLoS medicine. 2021 Jul 12;18(7):e1003714.
39. Essman M, Bishop T, Burgoine T, Jones A, Polden M, Robinson E, Sharp S, Smith R, White M, Adams J. OP81 Experiences and process of implementation and enforcement of mandatory calorie labelling regulations for the out-of-home sector in England: a qualitative study.
40. Putra IG, Polden M, Wareing L, Robinson E. Acceptability and perceived harm of calorie labelling and other obesity policies: a cross-sectional survey study of UK adults with eating disorders and other mental health conditions.

For peer review only

Supplementary Materials

Table of contents

1. Section 1: List of Standard Industrial Classification codes used for sampling 2
2. Section 2: Full list of businesses assessed for kcal labelling practices 3
3. Section 3: Measures used to assess kcal labelling based on government guidance 4
4. Section 4: Power Analysis 5
5. Section 5: Supplementary Tables 6

For peer review only

Section 1: List of Standard Industrial Classification codes used for sampling

Large businesses with the following Standard Industrial Classification (SIC) codes (and hence in the following IDBR sections) that are subject to the policy and thus included in this study:

Section I (accommodation and food service activities)
Within section G:
- SIC 47.11- Retail sale in non-specialised stores with food, beverages or tobacco predominating.
- SIC 47.24- Retail sale of bread, cakes, flour confectionery and sugar confectionery in specialised stores.
- SIC 47.29 Other retail sales of food in specialised stores.

Within section R:
- SIC 91.03-Operation of historical sites and buildings and similar visitor attractions
- SIC 91.04-Botanical and zoological gardens and nature reserve
- SIC 93.11-Operation of sports facilities
- SIC 93.12-Activities of sports clubs
- SIC 93.13-Fitness facilities
- SIC 93.21-Activities of amusement parks and theme parks

Within section J:
- SIC 59.14-Motion picture projection activities

For peer review only

Section 2: Full list of businesses assessed for kcal labelling practices

ASK
Beefeater Grill
Bella Italia
Bespoke Hotels Ltd
Bills
Brasserie Blanc
Brewdog
Brewers Fayre
Britannia Adelphi Hotel
Burger King
Byron
Caffe Nero
Campanile Hotels
Charles Wells Pub Company
Chiquito
Cineworld
Costa
Cote
Crystal Leisure Ltd
Dudley zoo
Ember Inns
Fayre-square
Fish n chicken
Five Guys
Franco Manca
Frankie & Benny's
Fuller's
Gourmet Burger Kitchen
Greene King
Hallmark Hotel Group
Harris and Hoole
Harvester
Hickory's Smokehouse
Hilton Hotels & Resorts
Holiday Inn
Joe & The Juice
John Lewis
Kew Gardens
KFC
La Tasca
Las iguana
Le Bistrot Pierre
Leon
M & S
Marriott
Marston’s
McDonald's
Mercure
Millennium & Copthorne Hotels
Miller & Carter
Mitchells & Butler
Morrisons Daily
Nando’s
Nicholson's Pubs
Oak Tree Pubs
Odeon Cinemas
Pitcher and piano
Pizza Express
Pizza Hut
Premier Inn
Radisson Blu
Reel Cinemas
Revolution
S A Brain
Sainsbury's
Shepherd Neame Pubs
Showcase Cinemas
Sizzling Pub
Slug & Lettuce
Soho Coffee Co.
Starbucks Coffee
Stone house restaurants
Stonegate Pubs
Subway
T G I Friday's
Tesco
The Ivy Cafe
The Talbot Hotel
Tortilla
Travelodge
Turtle Bay
Vintage Inns
Wagamama
Walker Art Gallery
Wetherspoon
white 4brasserie
Yee Rah
YO SUSHI
Young's
Zizzi

For peer review only

Section 3: Measures used to assess kcal labelling practices based on government guidance (DHSC, 2018).

Kcal labelling Criteria	Rating	
1) Is kcal labelling provided at any point of choice? (e.g menus, display board)	Y	N
2) Is kcal labelling provided at all points of choice? (Menus, display boards etc.)	Y	N
3) Is kcal labelling provided for all eligible food items?	Y	N
4) Is kcal labelling provided for all non-alcoholic drink items?	Y	N
5) Is kcal labelling provided per portion for shareable items?	Y	N
6) Is kcal labelling presented close to the item's name or price?	Y	N
7) Is kcal labelling presented as prominently as name or price?	Y	N
8) Is kcal reference info for an adult woman displayed anywhere?	Y	N
9) Is kcal reference info displayed clearly and prominently?	Y	N

For peer review only

Section 4: Power Analysis

The sample size was determined based on 2018 data showing that 17% of large OHFO in the UK provided voluntary kcal labelling (Robinson et al, 2018). Based on a power calculation a minimum sample size of n=96 (24 outlets per local authority) at pre and post implementation would give 80% power to detect a doubling of prevalence from 17% to 34% at α=0.05, (assuming a pre-post correlation of 0.1). We therefore aimed to recruit a minimum of 96 outlets at pre and post implementation.

For peer review only

Section 5: Supplementary Tables

Table 1: Effects of labelling regulations (pre-post), index of multiple deprivation, local authority and outlet type on presentation of any kcal labelling in unique outlets.

	**Any kcal labelling**		
Exposure variables	*Odds Ratios*	*99% CI*	*p*
Time (post-regulations)	25.64	3.72 – 176.87	**<0.001**
IMD-2	0.41	0.06 – 2.92	0.241
IMD-3	1.23	0.20 – 7.41	0.771
IMD-4	0.15	0.01 – 2.87	0.097
IMD-5	0.32	0.03 – 3.31	0.210
LA Liverpool	0.83	0.14 – 5.01	0.790
LA Milton Keynes	0.83	0.14 – 4.93	0.787
LA Richmond	1.42	0.15 – 13.84	0.690
Outlet Type (entertainment, retail, attraction)	0.62	0.10 – 3.87	0.498
Outlet Type (restaurants)	0.48	0.12 – 1.89	0.169
**Random Effects**			
σ^2	3.29		
$\tau_{00\ outletid}$	1.08		
ICC	0.25		
$N_{outletid}$	90		
Marginal R^2 / Conditional R^2	0.410 / 0.566		

Legend: IMD = Index of multiple deprivation; LA = Local authority. Reference categories were IMD = 1; Local authority of Dudley; cafes, fast-food, pubs and hotels for outlet type.

For peer review only

Table 2: Effects of labelling regulations, index of multiple deprivation, local authority and outlet type on total compliance score for kcal labelling for unique outlets.

	**Total Compliance Score (0-9)**		
Exposure variables	*Incidence Rate Ratios*	*99% CI*	*p*
Time (post-regulations)	5.19	3.83 – 7.04	**<0.001**
IMD-2	0.79	0.38 – 1.65	0.408
IMD-3	1.11	0.57 – 2.16	0.687
IMD-4	0.50	0.16 – 1.52	0.108
IMD-5	0.74	0.32 – 1.71	0.351
LA Liverpool	0.83	0.42 – 1.62	0.464
LA Milton Keynes	0.86	0.45 – 1.67	0.567
LA Richmond	1.06	0.45 – 2.47	0.862
Outlet Type (entertainment, retail, attraction)	0.83	0.42 – 1.66	0.494
Outlet Type (restaurants)	0.89	0.54 – 1.45	0.527
**Random Effects**			
σ^2	0.37		
$\tau_{00\ \text{outletid}}$	0.45		
ICC	0.55		
N_{outletid}	90		
Marginal R^2 / Conditional R^2	0.470 / 0.760		

Legend: IMD = Index of multiple deprivation; LA = Local authority. Reference categories were IMD = 1; Local authority of Dudley; cafes, fast-food, pubs and hotels for outlet type.

For peer review only

Table 3: Interactions between time and index of multiple deprivation for compliance total score.

	Compliance Total Score (0-9)		
Exposure variables	*Incidence Rate Ratios*	*99 % CI*	*p*
Time (post-regulations)	3.68	2.52 – 5.38	**<0.001**
IMD-2	0.05	0.00 – 0.67	**0.003**
IMD-3	1.35	0.69 – 2.63	0.244
IMD-4	0.22	0.04 – 1.09	0.015
IMD-5	0.30	0.13 – 0.69	**<0.001**
Time * IMD-2	18.73	1.42 – 246.54	**0.003**
Time * IMD-3	0.74	0.41 – 1.37	0.211
Time * IMD-4	3.44	0.72 – 16.44	0.042
Time * IMD-5	2.97	1.33 – 6.62	**<0.001**
**Random Effects**			
σ^2	0.33		
$\tau_{00\ outletid}$	0.31		
ICC	0.49		
$N_{outletid}$	114		
Observations	228		
Marginal R^2 / Conditional R^2	0.713 / 0.853		

Legend: IMD = Index of multiple deprivation; LA = Local authority. Reference categories were IMD = 1.

For peer review only

Table 4: Interactions between time and local authority for compliance total score.

	Compliance Total Score (0-9)		
Exposure variables	*Incidence Rate Ratios*	*99% CI*	*p*
Time (post-regulations)	6.25	3.58 – 10.91	**<0.001**
LA Liverpool	1.34	0.61 – 2.98	0.342
LA Milton Keynes	1.28	0.58 – 2.81	0.427
LA Richmond	0.80	0.33 – 1.89	0.496
Time * LA Liverpool	0.69	0.33 – 1.43	0.189
Time * LA Milton Keynes	0.65	0.31 – 1.36	0.135
Time * LA Richmond	1.05	0.47 – 2.37	0.874
**Random Effects**			
σ^2	0.33		
$\tau_{00\ \text{outletid}}$	0.35		
ICC	0.51		
N_{outletid}	114		
Observations	228		
Marginal R^2 / Conditional R^2	0.511 / 0.762		

Legend: LA = Local authority. Reference categories were local authority of Dudley.

For peer review only

Table 5: Interactions between time and outlet type for compliance total score.

	Compliance Total Score (0-9)		
Exposure variables	*Incidence Rate Ratios*	*CI*	*p*
Time (post-regulations)	3.52	2.57 – 4.82	**<0.001**
Outlet Type (entertainment, retail, attraction)	1.22	0.58 – 2.57	0.501
Outlet Type (restaurants)	0.11	0.04 – 0.34	**<0.001**
Time * Outlet Type (entertainment, retail, attraction)	0.68	0.35 – 1.35	0.148
Time * Outlet Type (restaurants)	10.66	3.58 – 31.78	**<0.001**
**Random Effects**			
σ^2	0.33		
$\tau_{00\ outletid}$	0.35		
ICC	0.52		
$N_{outletid}$	114		
Observations	228		
Marginal R^2 / Conditional R^2	0.711 / 0.860		

Legend: Reference categories were cafes, fast-food, pubs and hotels for outlet type.

For peer review only

STROBE Statement—checklist of items that should be included in reports of observational studies

	Item No	Recommendation	Page No
**Title and abstract**	1	(*a*) Indicate the study's design with a commonly used term in the title or the abstract	1
		(*b*) Provide in the abstract an informative and balanced summary of what was done and what was found	2
**Introduction**			
Background/rationale	2	Explain the scientific background and rationale for the investigation being reported	4-5
Objectives	3	State specific objectives, including any prespecified hypotheses	6
**Methods**			
Study design	4	Present key elements of study design early in the paper	7
Setting	5	Describe the setting, locations, and relevant dates, including periods of recruitment, exposure, follow-up, and data collection	6-7
Participants	6	(*a*) *Cohort study*—Give the eligibility criteria, and the sources and methods of selection of participants. Describe methods of follow-up<br>*Case-control study*—Give the eligibility criteria, and the sources and methods of case ascertainment and control selection. Give the rationale for the choice of cases and controls<br>*Cross-sectional study*—Give the eligibility criteria, and the sources and methods of selection of participants	n/a
		(*b*) *Cohort study*—For matched studies, give matching criteria and number of exposed and unexposed<br>*Case-control study*—For matched studies, give matching criteria and the number of controls per case	n/a
Variables	7	Clearly define all outcomes, exposures, predictors, potential confounders, and effect modifiers. Give diagnostic criteria, if applicable	8-9
Data sources/ measurement	8*	For each variable of interest, give sources of data and details of methods of assessment (measurement). Describe comparability of assessment methods if there is more than one group	*n/a*
Bias	9	Describe any efforts to address potential sources of bias	8
Study size	10	Explain how the study size was arrived at	7
Quantitative variables	11	Explain how quantitative variables were handled in the analyses. If applicable, describe which groupings were chosen and why	8-9
Statistical methods	12	(*a*) Describe all statistical methods, including those used to control for confounding	8-9
		(*b*) Describe any methods used to examine subgroups and interactions	8-9
		(*c*) Explain how missing data were addressed	8-9
		(*d*) *Cohort study*—If applicable, explain how loss to follow-up was addressed<br>*Case-control study*—If applicable, explain how matching of cases and controls was addressed<br>*Cross-sectional study*—If applicable, describe analytical methods taking account of sampling strategy	8-9
		(*e*) Describe any sensitivity analyses	8-9

Continued on next page

For peer review only

**Results**			
Participants	13*	(a) Report numbers of individuals at each stage of study—eg numbers potentially eligible, examined for eligibility, confirmed eligible, included in the study, completing follow-up, and analysed	n/a
		(b) Give reasons for non-participation at each stage	
		(c) Consider use of a flow diagram	
Descriptive data	14*	(a) Give characteristics of study participants (eg demographic, clinical, social) and information on exposures and potential confounders	n/a
		(b) Indicate number of participants with missing data for each variable of interest	n/a
		(c) *Cohort study*—Summarise follow-up time (eg, average and total amount)	11
Outcome data	15*	*Cohort study*—Report numbers of outcome events or summary measures over time	*n/a*
		Case-control study—Report numbers in each exposure category, or summary measures of exposure	
		Cross-sectional study—Report numbers of outcome events or summary measures	
Main results	16	(*a*) Give unadjusted estimates and, if applicable, confounder-adjusted estimates and their precision (eg, 95% confidence interval). Make clear which confounders were adjusted for and why they were included	12-15
		(*b*) Report category boundaries when continuous variables were categorized	n/a
		(*c*) If relevant, consider translating estimates of relative risk into absolute risk for a meaningful time period	n/a
Other analyses	17	Report other analyses done—eg analyses of subgroups and interactions, and sensitivity analyses	15-16
**Discussion**			
Key results	18	Summarise key results with reference to study objectives	16-18
Limitations	19	Discuss limitations of the study, taking into account sources of potential bias or imprecision. Discuss both direction and magnitude of any potential bias	19
Interpretation	20	Give a cautious overall interpretation of results considering objectives, limitations, multiplicity of analyses, results from similar studies, and other relevant evidence	20
Generalisability	21	Discuss the generalisability (external validity) of the study results	19-20
**Other information**			
Funding	22	Give the source of funding and the role of the funders for the present study and, if applicable, for the original study on which the present article is based	20

*Give information separately for cases and controls in case-control studies and, if applicable, for exposed and unexposed groups in cohort and cross-sectional studies.

Note: An Explanation and Elaboration article discusses each checklist item and gives methodological background and published examples of transparent reporting. The STROBE checklist is best used in conjunction with this article (freely available on the Web sites of PLoS Medicine at http://www.plosmedicine.org/, Annals of Internal Medicine at http://www.annals.org/, and Epidemiology at http://www.epidem.com/). Information on the STROBE Initiative is available at www.strobe-statement.org.

For peer review only

For peer review only - http://bmjopen.bmj.com/site/about/guidelines.xhtml

Point-of-choice kilocalorie labelling practices in large, out-of-home food businesses: A pre vs post observational study of labelling practices following implementation of kilocalorie Labelling (Out of Home Sector) (England) Regulations 2021

Journal:	*BMJ Open*
Manuscript ID	bmjopen-2023-080405.R1
Article Type:	Original research
Date Submitted by the Author:	16-Feb-2024

Complete List of Authors:	Polden, Megan; University of Liverpool, Department of Primary Care and Mental Health; Lancaster University<br>Jones, Andrew; Liverpool John Moores University<br>Essman, Michael; University of Cambridge, MRC Epidemiology Unit<br>Adams, Jean; University of Cambridge, MRC Epidemiology Unit<br>Bishop, Tom; University of Cambridge, MRC Epidemiology Unit<br>Burgoine, Thomas; University of Cambridge, MRC Epidemiology Unit<br>Donohue, Aisling; Liverpool John Moores University<br>Sharp, Stephen; University of Cambridge, MRC Epidemiology Unit<br>White, Martin; University of Cambridge, MRC Epidemiology Unit<br>Smith, Richard ; University of Exeter Medical School, Institute of Health Research<br>Robinson, Eric; University of Liverpool, Department of Psychology

**Primary Subject Heading**:	Public health
Secondary Subject Heading:	Health policy, Nutrition and metabolism, Public health
Keywords:	Nutrition < TROPICAL MEDICINE, Public health < INFECTIOUS DISEASES, Health policy < HEALTH SERVICES ADMINISTRATION & MANAGEMENT, Obesity

I, the Submitting Author has the right to grant and does grant on behalf of all authors of the Work (as defined in the below author licence), an exclusive licence and/or a non-exclusive licence for contributions from authors who are: i) UK Crown employees; ii) where BMJ has agreed a CC-BY licence shall apply, and/or iii) in accordance with the terms applicable for US Federal Government officers or employees acting as part of their official duties; on a worldwide, perpetual, irrevocable, royalty-free basis to BMJ Publishing Group Ltd ("BMJ") its licensees and where the relevant Journal is co-owned by BMJ to the co-owners of the Journal, to publish the Work in this journal and any other BMJ products and to exploit all rights, as set out in our licence.

The Submitting Author accepts and understands that any supply made under these terms is made by BMJ to the Submitting Author unless you are acting as an employee on behalf of your employer or a postgraduate student of an affiliated institution which is paying any applicable article publishing charge ("APC") for Open Access articles. Where the Submitting Author wishes to make the Work available on an Open Access basis (and intends to pay the relevant APC), the terms of reuse of such Open Access shall be governed by a Creative Commons licence – details of these licences and which Creative Commons licence will apply to this Work are set out in our licence referred to above.

Other than as permitted in any relevant BMJ Author's Self Archiving Policies, I confirm this Work has not been accepted for publication elsewhere, is not being considered for publication elsewhere and does not duplicate material already published. I confirm all authors consent to publication of this Work and authorise the granting of this licence.

For peer review only

Title: Point-of-choice kilocalorie labelling practices in large, out-of-home food businesses: A pre vs post observational study of labelling practices following implementation of kilocalorie Labelling (Out of Home Sector) (England) Regulations 2021

For peer review only

Author names and affiliations:

Megan Polden, Department of Primary Care and Mental Health, University of Liverpool

Andrew Jones, Aisling Donohue, Liverpool John Moores University

Michael Essman, Jean Adams, Tom Bishop, Thomas Burgoine, Stephen J. Sharp, Martin White, MRC Epidemiology Unit, University of Cambridge

Richard Smith, Faculty of Health and Life Sciences, University of Exeter

Eric Robinson, Department of Psychology, University of Liverpool

Corresponding author information: Megan Polden, Department of Primary Care and Mental Health, University of Liverpool, Liverpool, UK, m.polden@liverpool.ac.uk

Abstract

Background and Objectives: On 6th April 2022 the UK government implemented mandatory kilocalorie (kcal) labelling regulations for food and drink products sold in the out-of-home food sector (OHFS) in England. Previous assessments of kcal labelling practices in the UK OHFS found a low prevalence of voluntary implementation and poor compliance with labelling recommendations. This study aimed to examine changes in labelling practices pre vs. post-implementation of mandatory labelling regulations in 2022.

Methods: In August-December 2021 (pre-implementation) and August-November 2022 (post-implementation), large OHFS businesses (250 or more employees) subject to labelling regulations were visited. At two time points a researcher visited the same 117 food outlets (belonging to 90 unique businesses) across four local authorities in England. Outlets were rated for compliance with government regulations for whether kcal labelling was: provided at any or all point-of-choice, provided for all eligible food and drink items, provided per portion for sharing items, if labelling was clear and legible and if kcal reference information was displayed.

Results: There was a significant increase (21% pre-implementation vs. 80% post-implementation, OR = 40.98 [95% CI: 8.08 to 207.74], $p<0.001$) in the proportion of outlets providing any kcal labelling at point-of-choice post-implementation. Only 15% of outlets met all labelling compliance criteria post-implementation, with a minority of outlets not presenting labelling in a clear (33%) or legible (29%) way.

Conclusion: The number of large businesses in the OHFS providing kcal labelling increased following the implementation of mandatory labelling regulations. However, around one-fifth of eligible outlets sampled were not providing kcal labelling 4-8 months after the regulations came into force, and the majority of businesses only partially complied with government

For peer review only

guidance. More effective enforcement may be required to further improve kcal labelling practices in the OHFS in England.

Registration: Study protocol and analysis strategy pre-registered on Open Science Framework (https://osf.io/pfnm6/).

Keywords: Kcal labelling, nutrition, public health, out-of-home food

For peer review only

Article Summary

Strengths and Limitations:

- This is the first study in England to investigate kcal labelling compliance in the out-of-home food sector pre vs. post-implementation of mandatory kcal labelling regulations.
- This study had high inter-rater reliability when examining adherence to labelling guidance and assessed a range of OHFS businesses from multiple local authorities sampled from across the socio-economic spectrum.
- The study only examined large OHFS businesses and therefore findings are not reflective of the whole OHFS
- The current study did not examine consistency across different outlets within a chain and therefore conclusions cannot be drawn on the level of consistency of kcal labelling within large chains.

For peer review only - http://bmjopen.bmj.com/site/about/guidelines.xhtml

Introduction

The prevalence of obesity has been steadily rising both globally and in the UK since 1980 [1,2], with 64% of adults and 38% of 10–11-year-olds in England classified as having overweight or obesity [3]. Between 25% and 39% of UK adults reported eating food from the out-of-home food sector (OHFS) at least once per week in 2008-12 and 2018 [4-6].

Food purchased from the OHFS is often high in energy [7,8] and frequent consumption of this food is associated with poorer diet quality and overweight/obesity [4,9,10]. A 2021 study in England found that on average people purchased 1013 kcals per meal when eating out of the home and consumed an average of 915 kcals [11], significantly higher than the UK government recommendation of 600 kcals or less for a lunch or evening meal [12]. Consumers also tend to underestimate the energy content of food purchased from the OHFS [13,14]. In addition, poor-quality diet and obesity are associated with a lower socioeconomic position (SEP) [15] and people of lower SEP are more likely to report being less motivated by weight management or healthiness of food choices [16], which may contribute to SEP-related differences in OHFS behaviour.

Multiple countries and regions, including the US [17] and Canada [18], have introduced mandatory policies that require OHFS outlets to provide consumers with point-of-choice kcal labelling for food and drink products. Meta-analytic evidence from randomised control trials and longitudinal studies indicates that kcal labelling may have small public health benefits, as the provision of kcal labelling has been shown to reduce the number of kcals purchased in food outlets [19]. Consistent with this, a recent US study examining purchasing transaction data found that kcal labels were associated with approximately 25 fewer kcals purchased suggesting that labelling in real-world setting may influence consumer behaviour [20]. However, other reviews have concluded that kcal labelling has a negligible effect on consumer behaviour [21]. Effects are likely mediated to some extent by the quality

For peer review only

of labelling implemented and these labels being noticed and used. Thus, high-quality implementation will likely be important to maximise the effects of kcal labelling on consumer purchasing and consumption.

In 2011, the UK government launched the public health responsibility deal with companies making voluntary pledges to improve public health – including providing kcal labelling in the OHFS and best practice guidelines on how to provide labelling [22]. The extent to which this pledge resulted in business practice change was unclear, as evaluations were reliant on self-reported and incomplete data from businesses [23]. In a 2018 study, only 18 out of 104 (17%) unique business OHFS outlets in England provided in-store kcal labelling [23]. Furthermore, the quality of this kcal labelling was inconsistent, often lacking prominence and clarity, and not presented on all available food and drink items [24]. The overall quality of labelling presented is likely to impact whether customers notice and use kcal labelling when selecting products [25].

Motivated by the lack of voluntary kcal labelling compliance in the OHFS, the UK government consulted on mandating kcal labelling in 2018. Following public consultation [26], legislation was passed in July 2021, with mandatory kcal labelling in large food businesses required as of 6th April 2022 [27,28]. The regulations require large (250 or more employees) businesses (cafes, fast-food outlets, sit-down restaurants, pubs) in England selling food and non-alcoholic drinks in scope of the regulations to: (1) display the energy content of food in kcals; (2) reference to the size of the portion to which the kcal information relates (for example, sharing portion for two people); and (3) display the statement that 'adults need around 2000 kcal a day'. Food and non-alcoholic drinks are in scope of the regulations and classed as eligible if they are: (1) offered for sale in a form which is suitable for immediate consumption; (2) not prepacked food; and (3) not exempt food such as food on a temporary menu for less than 30 days [29]. The regulations state that labelling should be easily visible,

For peer review only

clearly legible and not in any way hidden or obscured. The labelling must be displayed on the menu next to the description or price of the food. If food is on display in a food counter the labelling must be next to or in close proximity to each food item and displayed in a position which ensures that the label can be read by the customer [29].

To date, only one study has examined OHFS adherence to mandatory kcal labelling legislation [30]. This US study found an overall high level of compliance (94%) across 197 chain restaurants subject to federal menu labelling law (20 or more US sites). Similar data is not currently available following the implementation of regulations in England and evidence on how OHFS kcal labelling practices changed following the implementation of mandatory kcal labelling will be able to inform any future development of the regulations.

This study aimed to compare kcal labelling practices in large OHFS businesses in England subject to kcal labelling regulations before (2021) and after (2022) mandatory labelling regulations in 2022. We examined changes in the proportion of outlets providing kcal labelling and the level of compliance with government guidelines regarding the clarity, prominence and legibility of the labelling; overall and by type of outlet and local authority deprivation.

Methods

Area and Outlet Sampling

Sampling of outlets was conducted in four purposively selected local government areas (i.e. local authorities (LA)) that ensured geographical coverage and representation across quintiles of deprivation (Index of Multiple Deprivation (IMD) [31] used at the local authority level). Local authority average IMD levels (1-5) were used with IMD1 reflecting the most deprived areas and IMD5 reflecting the least deprived, defined at the lower layer super output area to better capture small area geographic variations in IMD. The four LAs sampled were Liverpool

For peer review only

(IMD1 northern region), Dudley (IMD2 midlands), Milton Keynes (IMD3, South) and Richmond upon Thames (IMD5 London).

The Inter-Department Business Register [32] was used to identify eligible businesses likely to be subject to the mandatory kcal labelling policy within local authorities of interest (data sampled in June 2021, list produced in Autumn 2020). This is a list of all UK businesses, which includes their core characteristics, number of employees and principal activities defined using the Standard Industrial Classification. We identified Standard Industrial Classification codes likely to include businesses serving food (for the full list of Standard Industrial Classification codes we used see supplementary materials section 1) and then identified large businesses with 250 or more employees. Individual businesses often have multiple outlets (e.g. chain restaurants), so individual outlets belonging to each identified large business [33] within the four local authorities of interest were identified using Ordnance Survey Points of Interest [34] data from September 2020.

The sample size was determined based on 2018 data showing that 17% of large OHFOs in the UK provided voluntary kcal labelling [24]. Based on a power calculation (see online supplementary materials section 4 for full details), to detect a doubling of prevalence from 17% to 34%, we estimated we required a minimum sample size of 96 outlets. We used stratified sampling by LA and business type to sample a total of 117 outlets, representing 90 unique businesses, due to the limited availability of unique outlets in some local authorities. We prioritised sampling from unique business outlets (as opposed to sampling multiple outlets from the same business) as we assumed kcal practises would be more likely to differ between, as opposed to within, the same large businesses in the OHFS.

Three outlets closed in the post-policy period and were therefore excluded from the final analysis, resulting in a sample size of n=114 (87 unique businesses). This sample included outlets from attractions, cafes, fast food, hotels, pubs, restaurants, retail, and entertainment

For peer review only

businesses (full list of businesses in supplementary materials section 2) across the four local authorities.

Procedure

The kcal labelling assessment procedure was based on previous methods used to assess kcal labelling practises of large OHFS businesses in England during 2018 [24]. Researchers visited selected outlets in August-November 2021 (pre-policy) and August-November 2022 (post-policy) to examine if kcal labelling was present and whether it adhered to labelling guidelines provided by the Department of Health and Social Care [29,35,36]. In each outlet, researchers examined point-of-choice menu display boards inside and outside the outlet, handheld food and drink menus and menus presented at ordering points. A researcher rated the presence of any kcal labelling (0=no, 1=yes), as well whether kcal labelling adhered to the following criteria: (1) provided at any point-of-choice (2) provided at all points-of-choice, (3) provided for all eligible food items, (4) provided per portion for sharing items, (5) presented close to the item's name and price, (6) presented as prominently as name or price, (7) provided alongside any kcal reference information, (8) provided alongside clearly and prominently displayed kcal reference information, (9) provided for all non-alcoholic drink items (See Table 1). Eligible food and drink items include non-prepacked food that are suitable for immediate consumption. Exemptions include items on the menu for less the 30 days and drinks containing more than 1.2% by volume of alcohol [28].

A score between 0-9 was calculated for each outlet based on the number of kcal labelling practices the outlet adhered to. For example, a score of 3 would mean that the outlet adhered to 3 of the labelling guidelines (list of criteria in supplementary materials section 3). For the post-implementation assessment, three additional kcal labelling criteria relating to whether labelling was clearly presented, legible and whether a kcal-free menu was available

For peer review only

on request were added for further descriptive information (Table 1) based on kcal labelling guidance introduced alongside the new legislation [29].

Researchers received training on assessing outlet adherence to labelling guidelines and the study protocol before the start of data collection to ensure consistency between raters. Ten per cent (n=17) of outlets were randomly selected across all outlet locations and were independently coded by a second researcher with a percentage agreement score of 96% across all nine variables for the pre-assessment and 97% across all 12 variables for the post-assessment.

For peer review only

Patient and Public Involvement

This study did not include patient and public involvement.

Data Analysis

A descriptive overview is presented for kcal labelling practices pre and post-implementation by outlet type and IMD quintile of the postcode of the location at the lower layer super output area [37] (LSOA) level. Due to the small number of outlets for some outlet types (e.g. Entertainment and Attractions), we combined categories into three meaningful categories with adequate numbers: Restaurants; Entertainment, Retail and Attractions; Cafes, Fast-Food, Pubs, and Hotels.

To examine whether kcal labelling practices changed from before to after implementation and whether outlet characteristics were associated with kcal labelling regulations and compliance, regression models (logistic and Poisson) were used. Exposure variables were time (pre vs. post-implementation), outlet type (entertainment and retail venues; cafes, fast-food and pubs; and restaurants), deprivation level (LSOA IMD 1-5 (categorical)) and local authority (Liverpool, Dudley, Milton Keynes, Richmond Upon Thames). Where the

outcome variable was kcal labelling implemented in any form (yes/no), a logistic regression model was used. Where the outcome variable was the total compliance score (0-9), a Poisson regression model was used. Unadjusted models included only the time variable. Adjusted models additionally included outlet type, deprivation level (IMD of LSOA) and local authority. We repeated these analyses limiting them to unique outlets (see supplementary materials, section 5, tables 1 and 2). In instances where data was available from multiple outlets within a business, compliance data was used from the first outlet in which data was collected.

In subsequent pre-registered exploratory models, we planned to examine whether changes in compliance following labelling regulations differed by outlet type, local authority and deprivation level. However, some of these three-way interaction analyses were not possible due to small sample sizes (Ns < 5, e.g. only 2 restaurants had kcal information at baseline and only 4 entertainment/retail/attraction outlets did not report kcal information at post) meaning these interaction analyses would have been severely underpowered [38]. However, we conducted simple interaction analyses between time and IMD (at both IMD of LSOA and neighbourhood of outlet level), and time and outlet type for total compliance scores.

Finally, for the subset of outlets that had kcal labelling present at the pre-assessment, we performed the analysis described above (excluding interactions) to examine whether labelling regulations were associated with improvements in labelling quality among outlets already providing kcal labelling. This approach supplements the main analyses, by addressing improvements in labelling quality among existing compliant outlets, as changes in overall labelling quality in the primary analyses may be driven by outlets introducing labelling (as opposed to also increasing compliance with guidelines for how labelling is provided).

To account for the relatively large number of analyses conducted, confidence intervals and p-values were set to 99% and < .01 respectively to determine statistical significance. R and R Studio version 1.2.503 was used to conduct analyses, using the 'lme4', 'lmertest', 'sjPlot',

For peer review only

'descr' and 'performance' packages. Code and data are available here [https://osf.io/gp3rf/]. The study did not involve human participants and made use of publicly available information, therefore ethical approval was not required. The study protocol and analysis strategy were pre-registered on Open Science Framework (https://osf.io/pfnm6/).

Results

Table 1 shows adherence to the policy pre-and post-implementation of the regulations. 91/114 outlets (80%) provided kcal labelling at any point of choice post-implementation, an increase compared to pre-implementation with 24/117 (21%). Post-implementation, outlets were most compliant with presenting kcal information close to the item's name and price (80%) and displaying kcal referencing information (73%). Outlets were least compliant with presenting labelling that is as prominent as name or price (7%) and providing per portion information for sharing menu items (19%).

For peer review only

Table 1. Level of compliance with kcal labelling guidelines in outlets pre-and post-implementation.

	Pre- and post-implementation assessments in all outlets[1] (n=117)		Pre- and post-implementation assessments limited to outlets with labelling present pre and post (n=24)	
	Pre-implementation assessment N (%)	Post-implementation assessment N (%)	Pre-implementation assessment N (%)	Post-implementation assessment N (%)
Kcal labelling provided at any point of choice	24 (21%)	91 (80%)	24 (100%)	24 (100%)
Kcal labelling provided at all points of choice	16 (14%)	76 (67%)	16 (67%)	22 (92%)
Kcal labelling provided for all food items	17 (15%)	80 (70%)	17 (70%)	16 (67%)
Kcal labelling provided per portion for sharing menu items	10 (9%)	19 (17%)	10 (42%)	7 (29%)
Kcal labelling presented close to the item's name and price	21 (18%)	91 (80%)	21 (88%)	22 (92%)
Kcal labelling presented as prominently[1] as name or price	0 (0%)	7 (6%)	0 (0%)	2 (8%)
Kcal reference information displayed anywhere	11 (9%)	83 (73%)	11 (45%)	21 (88%)
Kcal reference information displayed clearly and prominently	4 (3%)	51 (45%)	4 (17%)	12 (50%)
Kcal labelling provided for all non-alcoholic drink items	11 (9%)	79 (69%)	11 (45%)	21 (88%)
**Additional data only available for post-implementation assessment**				
Kcal labelling legible[2]		76 (67%)		19 (79%)
Kcal labelling presented clearly[3]		81 (71%)		19 (79%)
Non-kcal menu available on request		11 (12%)		0 (0%)

[1]Note: Pre-implementation total outlets = 117 individual outlets from across 90 unique businesses. Post-implementation total outlets= 114 individual outlets from across 87 unique businesses post-implementation.

[1] The prominence of the labelling was determined based on whether the labelling was of equal prominence as item name or price i.e. text size and colour
[2] The legibility was determined by whether the kcal labelling was clear enough to read i.e. font size, font type and colour of text
[3] The clarity of the labelling was determined based on the kcal labelling was easy to understandable i.e. multiple kcal options presented close together or multiple kcal options requiring the customer to do additional sums to determine the kcal content.

For peer review only

Outcome: Kcal labelling presented in any form.

An unadjusted model with time (pre-implementation vs. post-implementation) demonstrated a significant effect with OR = 40.98 [95% CI: 8.08 to 207.74], Z = 4.48, p < .001: Marginal $R^2 = 0.40$), indicating an increased odds of kcal labelling presented in any form post-implementation of about 41 times than seen pre-implementation.

In an adjusted model (Table 2) the effect of time remained significant. However, the IMD quintile of outlet location, outlet type and local authority were not significantly associated with any kcal labelling.

Table 2: Effects of labelling regulations, index of multiple deprivation, local authority and outlet type on presentation of any kcal labelling

	**Any kcal labelling**		
Exposure variables	*Odds Ratios*	*99% CI*	*p*
Time (post-implementation)	35.11	4.87 – 252.98	**<0.001**
IMD-2	0.24	0.03 – 1.76	0.065
IMD-3	1.17	0.23 – 5.86	0.804
IMD-4	0.17	0.02 – 1.88	0.058
IMD-5	0.21	0.02 – 1.89	0.067
LA Liverpool	1.43	0.27 – 7.65	0.580
LA Milton Keynes	0.76	0.15 – 3.79	0.658
LA Richmond	1.63	0.19 – 13.67	0.555
Outlet Type (entertainment, retail, attractions)	0.50	0.09 – 2.89	0.307
Outlet Type (restaurants)	0.49	0.15 – 1.65	0.130
**Random Effects**			
σ^2	3.29		
$\tau_{00\ \mathrm{outletid}}$	1.07		
ICC	0.24		

For peer review only

$N_{outletid}$	114
Marginal R^2 / Conditional R^2	0.465 / 0.596

Legend: IMD = Index of multiple deprivation; LA = Local authority. Reference categories were pre-implementation, IMD = 1; Local authority of Dudley; cafes, fast-food, pubs and hotels for outlet type.

There was some evidence of multi-collinearity between LA (VIF = 3.57) and IMD quintile of LSOA (VIF = 4.50). Removal of either variable did not affect the models. We repeated the analyses limiting them to unique outlets. The overall pattern of results was similar; post-implementation led to OR = 25.64 (95% CI: 3.72 – 176.87) increased odds for any kcal labelling, in the full model (see online supplementary materials section 5).

Overall compliance score

A multilevel Poisson model with a random intercept of outlet examined overall compliance score (0 – 9). An unadjusted model with time (pre-implementation vs. post-implementation) demonstrated a significant effect with IRR = 5.06 [95% CI: 3.90 to 6.56], Z = 16.10, $p < .001$: Marginal $R^2 = 0.49$), indicating that compliance score was 6 times greater post vs pre-implementation of the regulations. Pre-implementation, the mean compliance score was 1.00/9 (2.18) and post-implementation it was = 5.06/9 (2.80). In an adjusted model (Table 3) time remained significant and the IMD quintile at the LSOA level, outlet type and local authority were not significant predictors of the overall compliance score.

For peer review only

Table 3: Effects of labelling regulations, index of multiple deprivation, local authority and outlet type on total compliance score for kcal labelling

	**Total Compliance Score (0-9)**		
Exposure variables	*Incidence Rate Ratios*	*99% CI*	*p*
Time (post-implementation)	5.06	3.90 – 6.56	**<0.001**
IMD-2	0.70	0.38 – 1.28	0.128
IMD-3	1.10	0.67 – 1.80	0.623
IMD-4	0.61	0.29 – 1.29	0.088
IMD-5	0.66	0.34 – 1.29	0.110
LA Liverpool	0.99	0.59 – 1.65	0.948
LA Milton Keynes	0.85	0.51 – 1.40	0.395
LA Richmond	1.10	0.56 – 2.17	0.704
Outlet Type (entertainment, retail, attraction)	0.82	0.48 – 1.43	0.366
Outlet Type (restaurants)	0.94	0.65 – 1.36	0.650
**Random Effects**			
σ^2	0.33		
$\tau_{00\ outletid}$	0.30		
ICC	0.48		
$N_{outletid}$	114		
Marginal R^2 / Conditional R^2	0.528 / 0.754		

Legend: IMD = Index of multiple deprivation; LA = Local authority. Reference categories were pre-implementation, IMD = 1; Local authority of Dudley; cafes, fast-food, pubs and hotels for outlet type.

There was some evidence of multi-collinearity between local authority (VIF = 3.50) and IMD quintile (VIF = 3.50). Removal of either variable from the model did not

For peer review only

substantially influence the results. We repeated the analyses limiting them to only unique outlets. The overall pattern of results was similar; post-implementation led to IRR = 5.19 (95% CI: 3.83 – 7.04) increase in any kcal labelling, in the full model (see online supplementary materials section 5).

In separate models, we examined the interaction between time and IMD, time and LA, and time and outlet type. There were no significant interactions between time and LA (ps > .135). There was a significant interaction between time and outlet type, specifically restaurants (IRR = 10.66 [95% CI: 3.58 to 31.78], p < .001). Compared to cafès, fast-food, pubs and hotels, there were greater increases in compliance for kcal labelling in restaurants post-implementation (increase in compliance scores in restaurants = 5.33, increase in cafes, fast-food, pubs and hotels = 3.54). Finally, there was a significant interaction between time and IMD, specifically for IMD2 (IRR = 18.73 [95% CI: 1.42 – 246.54], p = .003) and IMD5 (IRR = 2.97 [95% CI: 1.33 to 6.62], p < .001). There was a larger increase in compliance scores for IMD2 (increase = 4.86) and IMD5 (increase = 4.34), compared to IMD1 (increase = 3.92). Full model reporting can be found in online supplementary section 5, tables 3-5.

Exploratory analysis: Examining changes in compliance score among 24 outlets that displayed kcal at both time periods

We limited our analysis to the 24 outlets which had any kcal labelling at pre-implementation and examined whether labelling regulations increased the total compliance score. There was no evidence that the total compliance scores significantly increased from pre to post-implementation in these outlets (IRR = 1.25 [95% CI: 0.97 – 1.59], z = 1.75, p = .081), indicating that the change in compliance score observed in the primary analysis was largely driven by more outlets providing any form of kcal labelling, as opposed to compliance to recommendations significantly increasing in outlets already providing kcal labelling. At the

For peer review only

pre-implementation time point mean compliance score =4.75/9 (2.17) and at post mean compliance score = 5.92/9 (2.28). Frequency data on individual labelling criteria pre vs. post for the subset of 24 outlets is presented in Table 1.

Discussion

Overall, results indicated that labelling regulations were associated with a significant increase in the likelihood of OHFS outlets providing kcal labelling, with 21% in 2021 compared to 80% in 2022 providing labelling in any form. Compliance with government guidelines for nine kcal labelling criteria also increased post-implementation. The changes indicate the policy had a significant impact on labelling practices and prevalence in the OHFS. However, it should be noted that although compliance overall increased, there was still a substantial number of eligible outlets not providing any form of kcal labelling post-implementation of the policy (20%). In addition, there remained a lack of compliance with labelling guidelines, particularly in relation to presenting labelling and kcal reference information clearly and prominently; only 17/114 outlets (15%) met all guideline criteria post-implementation. When analyses were limited to outlets already implementing kcal labelling pre-legislation, there was no evidence of a change in compliance scores for labelling guidelines. This finding suggests that the increase in overall compliance scores from pre to post-implementation observed in the full sample was strongly driven by outlets introducing new labelling, as opposed to substantial improvements in the overall quality of existing labelling.

Twenty per cent of eligible outlets sampled were not providing any in-store kcal labelling at the time of post-implementation assessment. This reflects a rate of non-compliance greater than that observed in a US study, where only 6% of eligible outlets were not implementing kcal labelling in a similar number of unique businesses [30]. Of the outlets that provided in-store kcal labelling in our study, most were not presented as prominently as

For peer review only

name or price, presented at all points of choice, presented on all eligible food and drink items and for some outlets, it was not presented clearly or legibly. These results are consistent with research conducted prior to the announcement of the 2022 kcal labelling policy which found that existing voluntary kcal labelling did not align with government recommendations in the UK OHFS [24]. We also examined the legibility and clarity of kcal labelling post-regulations as this was specified in the legislation. The majority of outlets sampled post-regulations presented kcal labelling that was legible (67%) and clear (71%), but it often lacked prominence (94% of kcal labelling rated as not as prominent as name or price). This lack of compliance and potential hesitancy by food outlets to provide labelling information that is clear, legible and prominent may reflect an attempt by businesses to minimise the potential for kcal information to negatively impact product sales. Additionally, a lack of public support for kcal labelling policy [39] may have impacted on businesses willingness to fully comply with labelling guidance and in particular provide labelling that is prominent to customers. Food industries, specifically pizza chains in the US, have argued for flexibility when providing labelling due to difficulties reporting kcal amounts for customisable food items with multiple combinations and frequently changing menus [30], and this may be a contributory factor to a lack of compliance in England, particularly when reporting sharing options on menus. The level of impact of mandatory kcal labelling on consumer behaviour may be dependent on consumers' level of engagement [39] and this may be mediated by the prevalence and prominence of kcal labelling in the OHFS. Whether kcal labelling is implemented and labelling is clearly and prominently presented is likely to impact whether customers notice and use kcal labelling when selecting products [25].

There was a significant interaction between pre-post implementation and outlet type, specifically with restaurants showing a greater increase in compliance compared to cafes, fast-food, pubs and hotels. This interaction may have been driven by the particularly low

For peer review only

compliance levels of restaurants at the pre-implementation time point compared to other outlet types. Furthermore, there was a significant interaction between time and IMD, specifically that outlets from IMD quintiles 2 and 5 showed a statistically significant larger increase in compliance scores compared to IMD1. This may indicate that outlets located in more affluent areas showed greater improvement in kcal labelling practice post-regulations. Although it is not clear why this association was not found for IMD quintiles 3 and 4. The sample sizes in these analyses are small and further research examining the robustness of these associations is needed to determine their validity. This difference may also have been driven by the prevalence of different outlet types in each area, for example proportionally more fast-food establishments (as opposed to pubs) in Liverpool compared to Richmond Upon Thames.

Providing kcal information at the point of choice in the OHFS is hypothesised to support consumers in making informed dietary decisions and some, but not all, evidence indicates that this leads to consumers purchasing lower kcal and healthier food options [40]. As a proportion of major OHFS businesses are not complying with the kcal labelling regulations this may limit the impact on consumer behaviour.

Recent qualitative data suggested that local authorities are not engaging in pro-active enforcement, and instead relying on the presumed compliance of large businesses who they expect to comply with regulations to protect their reputations and maintain customer trust [41]. However, our study has found that 20% of eligible outlets are not adhering to kcal labelling regulations in any form and 85% are not complying with labelling guidance in full. Our findings suggest that greater enforcement by local authorities may be required to improve current labelling practices in the UK OHFS. It may also be the case that more specific details and regulations are needed in relation to how labelling should be presented within outlets, for example minimum font size, colour, font type. This may aid in improve the

For peer review only

legibility, clarity and prominence of labelling presented and also lead to greater consistency across businesses.

There has been some public concern about the implementation of mandatory kcal labelling in England [39], specifically about the potential negative impacts on people with eating disorders [42]. In an attempt to mitigate potential negative impacts, as part of government guidance for kcal labelling, it was recommended that kcal-free menus be available in outlets on request. Our results found that only 12% of outlets implementing kcal labelling were able to provide researchers with a kcal-free menu when requested in-store. Therefore, attempts to mitigate the potential harms of kcal labelling on vulnerable groups may require more formal guidance or a mandate to make the practice of kcal-free menus available on request more widespread.

Strengths and limitations

To our knowledge, this is the first study that has assessed and compared kcal labelling practices in the OHFS before and after the implementation of mandatory kcal labelling regulations in England. Data collection for kcal labelling assessments had high inter-rater reliability between researchers. This study assessed a range of OHFS businesses from multiple local authorities sampled from across the socio-economic spectrum in England, however, it is not clear how representative this sample is. As this study focused on large businesses subject to the kcal labelling policy, we did not examine kcal labelling practices in outlets exempt from the policy, including smaller businesses (<250 employees) and independent outlets, so this study does not give a full representation of kcal labelling practices across all businesses in the OHFS. Although encouraged, it is currently not mandatory for smaller OHFS businesses to implement kcal labelling. The present study provides data on those businesses where kcal labelling is mandated due to the legislation. It is therefore unclear whether the policy also impacted on kcal labelling practises in outlets

For peer review only

currently exempt from the legislation. Furthermore, it should be noted that exploratory analyses examining local area and outlet type variability in pre-post implementation changes to labelling were based on small sample sizes.

Additionally, this study examined labelling practices at a single time point post-implementation (approximately 4-8 months post-implementation) and it could be argued that some outlets needed more time to implement the regulations and comply with guidance. However, the government consulted on mandating kcal labelling in 2018 with a public consultation occurring in 2020. The legislation was then passed in 2021 with labelling being required from April 2022. This timeline of events provided businesses with ample time to prepare and enact required regulations. Therefore, it is unlikely that businesses did not have sufficient time to implement labelling regulations ahead of this labelling assessment and it is unlikely this is a legitimate reason for a lack of or poor compliance in some outlets in this assessment.

Future Research

Our study examined large businesses in the OHFS subject to mandatory kcal labelling regulations. There has been debate as to whether to expand these regulations to include medium and small OHFS businesses and future research could examine whether kcal labelling regulations also motivated smaller businesses not subject to the policy to provide voluntary kcal labelling on menus. Additionally, we were unable to examine whether labelling is provided consistently within outlets of the same business and this could be explored in future research. Research is also yet to examine the nutritional accuracy of newly implemented kcal labelling in the OHFS in England and future research would benefit from examining this.

Conclusions

For peer review only

Overall, the findings demonstrate a significant increase in kcal labelling practices in the OHFS in England following the implementation of the labelling regulations. However, there was still a substantial proportion of outlets not complying with the kcal labelling post-implementation, not fully meeting labelling guidance and providing kcal labelling in a way that undermines the likelihood of it being used by consumers. More proactive enforcement may be required to further improve kcal labelling practice in the UK OHFS and businesses should be encouraged to review their labelling practices to ensure continued compliance with labelling regulation guidelines.

Funding statement: This report is independent research commissioned and funded by the Department of Health and Social Care Policy Research Programme (Implementation and assessment of mandatory kcal labelling in the out-of-home sector, NIHR200689). The views expressed in this publication are those of the author(s) and not necessarily those of the NIHR or the Department of Health and Social Care. JA, TB, TB, ME, SS and MW are supported by the MRC Epidemiology Unit, University of Cambridge [UKRI grant number MC/UU/00006/7]. MP receives support from the NIHR Applied Research Collaboration ARC NWC and Alzheimer's Society and is funded through a Post-Doctoral Fellowship. ER is funded by the National Institute for Health and Care Research (NIHR) Oxford Health Biomedical Research Centre (BRC), Economic and Social Research Council (ES/W007932/1) and European Research Council (Grant reference: PIDS, 803194). The views expressed are those of the authors and not necessarily those of the funders, NHS or Department of Health and Social Care. For the purpose of Open Access, the author has applied a Creative Commons Attribution (CC BY) licence to any Author Accepted Manuscript version arising.

Competing interests statement: The authors declare no conflict of interest.

Author Contributions: Funding was obtained by J.A, E.R, A.J, M.W, R.S, T.B and S.S. Conceptualisation and methodology design contributions and project administration contributions were made by all authors. The formal analysis, data curation, and original draft

For peer review only

preparation contributions were made by M.P, E.R and A.J. All authors contributed to revising the manuscript. All authors read and agreed to the published version of the manuscript.

Availability of data and materials: Data from this study is available on the Open Science Framework (https://osf.io/gp3rf/).

References

1. Ng M, Fleming T, Robinson M, Thomson B, Graetz N, Margono C, Mullany EC, Biryukov S, Abbafati C, Abera SF, Abraham JP. Global, regional, and national prevalence of overweight and obesity in children and adults during 1980–2013: a systematic analysis for the Global Burden of Disease Study 2013. The lancet. 2014 Aug 30;384(9945):766-81.
2. Boutari C, Mantzoros CS. A 2022 update on the epidemiology of obesity and a call to action: as its twin COVID-19 pandemic appears to be receding, the obesity and dysmetabolism pandemic continues to rage on. Metabolism. 2022 Aug 1;133:155217.
3. Office for National Statistics. Obesity Profile: short statistical commentary. July 2022. https://www.gov.uk/government/statistics/obesity-profile-update-july-2022/obesity-profile-short-statistical-commentary-july-2022#further-information
4. Lachat C, Nago E, Verstraeten R, Roberfroid D, Van Camp J, Kolsteren P. Eating out of home and its association with dietary intake: a systematic review of the evidence. Obesity reviews. 2012 Apr;13(4):329-46.
5. Adams J, Goffe L, Brown T, Lake AA, Summerbell C, White M, Wrieden W, Adamson AJ. Frequency and socio-demographic correlates of eating meals out and take-away meals at home: cross-sectional analysis of the UK national diet and nutrition survey, waves 1–4 (2008–12). International Journal of Behavioral Nutrition and Physical Activity. 2015 Dec;12(1):1-9.
6. Mills S, Adams J, Wrieden W, White M, Brown H. Sociodemographic characteristics and frequency of consuming home-cooked meals and meals from out-of-home sources: cross-sectional analysis of a population-based cohort study. Public health nutrition. 2018 Aug;21(12):2255-66.
7. Roberts SB, Das SK, Suen VM, Pihlajamäki J, Kuriyan R, Steiner-Asiedu M, Taetzsch A, Anderson AK, Silver RE, Barger K, Krauss A. Measured energy content of frequently purchased restaurant meals: multi-country cross sectional study. bmj. 2018 Dec 12;363.
8. Robinson E, Jones A, Whitelock V, Mead BR, Haynes A. (Over) eating out at major UK restaurant chains: observational study of energy content of main meals. bmj. 2018 Dec 12;363.
9. Rosenheck R. Fast food consumption and increased caloric intake: a systematic review of a trajectory towards weight gain and obesity risk. Obesity reviews. 2008 Nov;9(6):535-47.
10. Taher AK, Evans N, Evans CE. The cross-sectional relationships between consumption of takeaway food, eating meals outside the home and diet quality in British adolescents. Public health nutrition. 2019 Jan;22(1):63-73.

11. Polden M, Jones A, Adams J, Bishop T, Burgoine T, Essman M, Sharp SJ, Smith R, White M, Robinson E. Kilocalorie labelling in the out-of-home sector: an observational study of business practices and consumer behaviour prior to implementation of the mandatory calorie labelling policy in England, 2022. BMC Public Health. 2023 Dec;23(1):1-2.
12. Public Health England. Calorie reduction: the scope and ambition. 2018. https://www.gov.uk/government/publications/calorie-reduction-the-scope-and-ambition-for-action.
13. Elbel B. Consumer estimation of recommended and actual calories at fast food restaurants. Obesity. 2011 Oct;19(10):1971-8.
14. Block JP, Condon SK, Kleinman K, Mullen J, Linakis S, Rifas-Shiman S, Gillman MW. Consumers' estimation of calorie content at fast food restaurants: cross sectional observational study. BMJ. 2013 May 23;346:f2907.
15. Adams J. Addressing socioeconomic inequalities in obesity: Democratising access to resources for achieving and maintaining a healthy weight. PLoS Medicine. 2020 Jul 28;17(7):e1003243.
16. Robinson E, Jones A, Marty L. The role of health-based food choice motives in explaining the relationship between lower socioeconomic position and higher BMI in UK and US adults. Int J Obes. 2022;21:1–7.
17. Cleveland LP, Simon D, Block JP. Compliance in 2017 with Federal Calorie Labeling in 90 chain restaurants and 10 retail food outlets prior to required implementation. American journal of public health. 2018 Aug;108(8):1099-102.
18. Goodman S, Vanderlee L, White CM, Hammond D. A quasi-experimental study of a mandatory calorie-labelling policy in restaurants: Impact on use of nutrition information among youth and young adults in Canada. Preventive medicine. 2018 Nov 1;116:166-72.
19. Crockett RA, King SE, Marteau TM, Prevost AT, Bignardi G, Roberts NW, Stubbs B, Hollands GJ, Jebb SA. Nutritional labelling for healthier food or non-alcoholic drink purchasing and consumption. Cochrane Database of Systematic Reviews. 2018(2).
20. Rummo PE, Mijanovich T, Wu E, Heng L, Hafeez E, Bragg MA, Jones SA, Weitzman BC, Elbel B. Menu Labeling and Calories Purchased in Restaurants in a US National Fast Food Chain. JAMA network open. 2023 Dec 1;6(12):e2346851.
21. Robinson E, Marty L, Jones A, White M, Smith R, Adams J. Will calorie labels for food and drink served outside the home improve public health?. Bmj. 2021 Jan 20;372.
22. Jebb SA. Calorie labelling on the high street. BMJ. 2011 Jul 26;343.
23. Knai C, Petticrew M, Durand MA, Eastmure E, James L, Mehrotra A, Scott C, Mays N. Has a public–private partnership resulted in action on healthier diets in England? An analysis of the Public Health Responsibility Deal food pledges. Food policy. 2015 Jul 1;54:1-0.
24. Robinson, E., Burton, S., Gough, T., Jones, A. and Haynes, A., 2019. Point of choice kilocalorie labelling in the UK eating out of home sector: a descriptive study of major chains. *BMC Public Health*, *19*, pp.1-6.
25. Nikolaou CK, Hankey CR, Lean ME. Calorie-labelling: does it impact on calorie purchase in catering outlets and the views of young adults?. International Journal of Obesity. 2015 Mar;39(3):542-5.

For peer review only - http://bmjopen.bmj.com/site/about/guidelines.xhtml

26. Department of Health & Social Care. Mandating calorie labelling in the out-of-home sector. Government response to public consultation. Mandating calorie labelling in the out-of-home sector: government response to public consultation (publishing.service.gov.uk).2020
27. Kaur A, Briggs A, Adams J, Rayner M. New calorie labelling regulations in England. bmj. 2022 May 4;377.
28. Department of Health & Social Care. Mandating calorie labelling in the out-of-home sector. 2020. https://assets.publishing.service.gov.uk/government/uploads/system/uploads/attachment_data/file/903714/Calorie_Labelling_-_Consultation_Response.pdf
29. Department of Health & Social Care. The Calorie Labelling (Out of Home Sector) (England) Regulations 2021. 2021. The Calorie Labelling (Out of Home Sector) (England) Regulations 2021 (https://www.legislation.gov.uk/uksi/2021/909/contents/made)
30. Cleveland LP, Simon D, Block JP. Federal calorie labelling compliance at US chain restaurants. Obesity Science & Practice. 2020 Apr;6(2):207-14.
31. National Statistics. English indices of deprivation. 2019. https://www.gov.uk/government/statistics/english-indices-of-deprivation-2019
32. Evans P, Welpton R. 'Business Structure Database: The Inter-Departmental Business Register (IDBR) for Research'. Economic & Labour Market Review. 2009;3(6):71-5.
33. Hughes JC, James G, Evans A, Prestwood D. Implementation of Standard Industrial Classification 2007: December 2009 update. Economic & Labour Market Review. 2009 Dec 1;3:51-5.
34. Points of interest: technical information. https://beta.ordnancesurvey.co.uk/products/points-of-interest
35. Department of Health. Technical Guidance on Voluntary Calorie Labelling for Catering Businesses. 2011. https://webarchive.nationalarchives.gov.uk/ukgwa/20180201183736/https://responsibilitydeal.dh.gov.uk/wp-content/uploads/2014/03/Technical-guidance-on-voluntary-calorie-labelling-DH-template-Updated.pdf
36. Food Standards Agency in Northern Ireland. Guidance on Voluntary Energy (kJ/KCAL) Labelling for Out of Home Businesses. 2017. https://acss.food.gov.uk/sites/default/files/caloriewisetechnicalguidance_1.pdf
37. Office for National Statistics. Rural urban classification (2011) of lower layer super output areas in England and Wales. 2011.
38. Leon AC, Heo M. Sample sizes required to detect interactions between two binary fixed-effects in a mixed-effects linear regression model. Computational statistics & data analysis. 2009 Jan 15;53(3):603-8.
39. Polden M, Robinson E, Jones A. Assessing public perception and awareness of UK mandatory calorie labelling in the out-of-home sector: Using Twitter and Google trends data. Obesity Science & Practice. 2022 Jul 11.
40. Petimar J, Zhang F, Rimm EB, Simon D, Cleveland LP, Gortmaker SL, Bleich SN, Polacsek M, Roberto CA, Block JP. Changes in the calorie and nutrient content of purchased fast food meals after calorie menu labeling: A natural experiment. PLoS medicine. 2021 Jul 12;18(7):e1003714.

41. Essman M, Bishop T, Burgoine T, Jones A, Polden M, Robinson E, Sharp S, Smith R, White M, Adams J. OP81 Experiences and process of implementation and enforcement of mandatory calorie labelling regulations for the out-of-home sector in England: a qualitative study.
42. Putra IG, Polden M, Wareing L, Robinson E. Acceptability and perceived harm of calorie labelling and other obesity policies: a cross-sectional survey study of UK adults with eating disorders and other mental health conditions.

For peer review only

Supplementary Materials

Table of contents

1. Section 1: List of Standard Industrial Classification codes used for sampling 2
2. Section 2: Full list of businesses assessed for kcal labelling practices 3
3. Section 3: Measures used to assess kcal labelling based on government guidance 4
4. Section 4: Power Analysis 5
5. Section 5: Supplementary Tables 6

For peer review only

Section 1: List of Standard Industrial Classification codes used for sampling

Large businesses with the following Standard Industrial Classification (SIC) codes (and hence in the following IDBR sections) that are subject to the policy and thus included in this study:

Section I (accommodation and food service activities)

Within section G:

- SIC 47.11- Retail sale in non-specialised stores with food, beverages or tobacco predominating.
- SIC 47.24- Retail sale of bread, cakes, flour confectionery and sugar confectionery in specialised stores.
- SIC 47.29 Other retail sales of food in specialised stores.

Within section R:

- SIC 91.03-Operation of historical sites and buildings and similar visitor attractions
- SIC 91.04-Botanical and zoological gardens and nature reserve
- SIC 93.11-Operation of sports facilities
- SIC 93.12-Activities of sports clubs
- SIC 93.13-Fitness facilities
- SIC 93.21-Activities of amusement parks and theme parks

Within section J:

- SIC 59.14-Motion picture projection activities

For peer review only

Section 2: Full list of businesses assessed for kcal labelling practices

ASK
Beefeater Grill
Bella Italia
Bespoke Hotels Ltd
Bills
Brasserie Blanc
Brewdog
Brewers Fayre
Britannia Adelphi Hotel
Burger King
Byron
Caffe Nero
Campanile Hotels
Charles Wells Pub Company
Chiquito
Cineworld
Costa
Cote
Crystal Leisure Ltd
Dudley zoo
Ember Inns
Fayre-square
Fish n chicken
Five Guys
Franco Manca
Frankie & Benny's
Fuller's
Gourmet Burger Kitchen
Greene King
Hallmark Hotel Group
Harris and Hoole
Harvester
Hickory's Smokehouse
Hilton Hotels & Resorts
Holiday Inn
Joe & The Juice
John Lewis
Kew Gardens
KFC
La Tasca
Las iguana
Le Bistrot Pierre
Leon
M & S
Marriott
Marston’s
McDonald's
Mercure
Millennium & Copthorne Hotels
Miller & Carter
Mitchells & Butler
Morrisons Daily
Nando’s
Nicholson's Pubs
Oak Tree Pubs
Odeon Cinemas
Pitcher and piano
Pizza Express
Pizza Hut
Premier Inn
Radisson Blu
Reel Cinemas
Revolution
S A Brain
Sainsbury's
Shepherd Neame Pubs
Showcase Cinemas
Sizzling Pub
Slug & Lettuce
Soho Coffee Co.
Starbucks Coffee
Stone house restaurants
Stonegate Pubs
Subway
T G I Friday's
Tesco
The Ivy Cafe
The Talbot Hotel
Tortilla
Travelodge
Turtle Bay
Vintage Inns
Wagamama
Walker Art Gallery
Wetherspoon
white 4brasserie
Yee Rah
YO SUSHI
Young's
Zizzi

For peer review only

Section 3: Measures used to assess kcal labelling practices based on government guidance (DHSC, 2018).

Kcal labelling Criteria	Rating	
1) Is kcal labelling provided at any point of choice? (e.g menus, display board)	Y	N
2) Is kcal labelling provided at all points of choice? (Menus, display boards etc.)	Y	N
3) Is kcal labelling provided for all eligible food items?	Y	N
4) Is kcal labelling provided for all non-alcoholic drink items?	Y	N
5) Is kcal labelling provided per portion for shareable items?	Y	N
6) Is kcal labelling presented close to the item's name or price?	Y	N
7) Is kcal labelling presented as prominently as name or price?	Y	N
8) Is kcal reference info for an adult woman displayed anywhere?	Y	N
9) Is kcal reference info displayed clearly and prominently?	Y	N

For peer review only

Section 4: Power Analysis

The sample size was determined based on 2018 data showing that 17% of large OHFO in the UK provided voluntary kcal labelling (Robinson et al, 2018). Based on a power calculation a minimum sample size of n=96 (24 outlets per local authority) at pre and post implementation would give 80% power to detect a doubling of prevalence from 17% to 34% at α=0.05, (assuming a pre-post correlation of 0.1). We therefore aimed to recruit a minimum of 96 outlets at pre and post implementation.

For peer review only

Section 5: Supplementary Tables

Table 1: Effects of labelling regulations (pre-post), index of multiple deprivation, local authority and outlet type on presentation of any kcal labelling in unique outlets.

	**Any kcal labelling**		
Exposure variables	*Odds Ratios*	*99% CI*	*p*
Time (post-regulations)	25.64	3.72 – 176.87	**<0.001**
IMD-2	0.41	0.06 – 2.92	0.241
IMD-3	1.23	0.20 – 7.41	0.771
IMD-4	0.15	0.01 – 2.87	0.097
IMD-5	0.32	0.03 – 3.31	0.210
LA Liverpool	0.83	0.14 – 5.01	0.790
LA Milton Keynes	0.83	0.14 – 4.93	0.787
LA Richmond	1.42	0.15 – 13.84	0.690
Outlet Type (entertainment, retail, attraction)	0.62	0.10 – 3.87	0.498
Outlet Type (restaurants)	0.48	0.12 – 1.89	0.169
**Random Effects**			
σ^2	3.29		
$\tau_{00\ \mathrm{outletid}}$	1.08		
ICC	0.25		
N_{outletid}	90		
Marginal R^2 / Conditional R^2	0.410 / 0.566		

Legend: IMD = Index of multiple deprivation; LA = Local authority. Reference categories were IMD = 1; Local authority of Dudley; cafes, fast-food, pubs and hotels for outlet type.

For peer review only

Table 2: Effects of labelling regulations, index of multiple deprivation, local authority and outlet type on total compliance score for kcal labelling for unique outlets.

	**Total Compliance Score (0-9)**		
Exposure variables	*Incidence Rate Ratios*	*99% CI*	*p*
Time (post-regulations)	5.19	3.83 – 7.04	**<0.001**
IMD-2	0.79	0.38 – 1.65	0.408
IMD-3	1.11	0.57 – 2.16	0.687
IMD-4	0.50	0.16 – 1.52	0.108
IMD-5	0.74	0.32 – 1.71	0.351
LA Liverpool	0.83	0.42 – 1.62	0.464
LA Milton Keynes	0.86	0.45 – 1.67	0.567
LA Richmond	1.06	0.45 – 2.47	0.862
Outlet Type (entertainment, retail, attraction)	0.83	0.42 – 1.66	0.494
Outlet Type (restaurants)	0.89	0.54 – 1.45	0.527
**Random Effects**			
σ^2	0.37		
$\tau_{00\ \mathrm{outletid}}$	0.45		
ICC	0.55		
N_{outletid}	90		
Marginal R^2 / Conditional R^2	0.470 / 0.760		

Legend: IMD = Index of multiple deprivation; LA = Local authority. Reference categories were IMD = 1; Local authority of Dudley; cafes, fast-food, pubs and hotels for outlet type.

For peer review only

Table 3: Interactions between time and index of multiple deprivation for compliance total score.

	Compliance Total Score (0-9)		
Exposure variables	*Incidence Rate Ratios*	*99 % CI*	*p*
Time (post-regulations)	3.68	2.52 – 5.38	**<0.001**
IMD-2	0.05	0.00 – 0.67	**0.003**
IMD-3	1.35	0.69 – 2.63	0.244
IMD-4	0.22	0.04 – 1.09	0.015
IMD-5	0.30	0.13 – 0.69	**<0.001**
Time * IMD-2	18.73	1.42 – 246.54	**0.003**
Time * IMD-3	0.74	0.41 – 1.37	0.211
Time * IMD-4	3.44	0.72 – 16.44	0.042
Time * IMD-5	2.97	1.33 – 6.62	**<0.001**
**Random Effects**			
σ^2	0.33		
$\tau_{00\ outletid}$	0.31		
ICC	0.49		
$N_{outletid}$	114		
Observations	228		
Marginal R^2 / Conditional R^2	0.713 / 0.853		

Legend: IMD = Index of multiple deprivation; LA = Local authority. Reference categories were IMD = 1.

For peer review only

Table 4: Interactions between time and local authority for compliance total score.

	**Compliance Total Score (0-9)**		
Exposure variables	*Incidence Rate Ratios*	*99% CI*	*p*
Time (post-regulations)	6.25	3.58 – 10.91	**<0.001**
LA Liverpool	1.34	0.61 – 2.98	0.342
LA Milton Keynes	1.28	0.58 – 2.81	0.427
LA Richmond	0.80	0.33 – 1.89	0.496
Time * LA Liverpool	0.69	0.33 – 1.43	0.189
Time * LA Milton Keynes	0.65	0.31 – 1.36	0.135
Time * LA Richmond	1.05	0.47 – 2.37	0.874
**Random Effects**			
σ^2	0.33		
$\tau_{00\ \mathrm{outletid}}$	0.35		
ICC	0.51		
N_{outletid}	114		
Observations	228		
Marginal R^2 / Conditional R^2	0.511 / 0.762		

Legend: LA = Local authority. Reference categories were local authority of Dudley.

For peer review only

Table 5: Interactions between time and outlet type for compliance total score.

	Compliance Total Score (0-9)		
Exposure variables	*Incidence Rate Ratios*	*CI*	*p*
Time (post-regulations)	3.52	2.57 – 4.82	**<0.001**
Outlet Type (entertainment, retail, attraction)	1.22	0.58 – 2.57	0.501
Outlet Type (restaurants)	0.11	0.04 – 0.34	**<0.001**
Time * Outlet Type (entertainment, retail, attraction)	0.68	0.35 – 1.35	0.148
Time * Outlet Type (restaurants)	10.66	3.58 – 31.78	**<0.001**
**Random Effects**			
σ^2	0.33		
$\tau_{00\ outletid}$	0.35		
ICC	0.52		
$N_{outletid}$	114		
Observations	228		
Marginal R^2 / Conditional R^2	0.711 / 0.860		

Legend: Reference categories were cafes, fast-food, pubs and hotels for outlet type.

For peer review only

STROBE Statement—checklist of items that should be included in reports of observational studies

	Item No	Recommendation	Page No
**Title and abstract**	1	(*a*) Indicate the study's design with a commonly used term in the title or the abstract	1
		(*b*) Provide in the abstract an informative and balanced summary of what was done and what was found	2
**Introduction**			
Background/rationale	2	Explain the scientific background and rationale for the investigation being reported	4-5
Objectives	3	State specific objectives, including any prespecified hypotheses	6
**Methods**			
Study design	4	Present key elements of study design early in the paper	7
Setting	5	Describe the setting, locations, and relevant dates, including periods of recruitment, exposure, follow-up, and data collection	6-7
Participants	6	(*a*) *Cohort study*—Give the eligibility criteria, and the sources and methods of selection of participants. Describe methods of follow-up<br>*Case-control study*—Give the eligibility criteria, and the sources and methods of case ascertainment and control selection. Give the rationale for the choice of cases and controls<br>*Cross-sectional study*—Give the eligibility criteria, and the sources and methods of selection of participants	n/a
		(*b*) *Cohort study*—For matched studies, give matching criteria and number of exposed and unexposed<br>*Case-control study*—For matched studies, give matching criteria and the number of controls per case	n/a
Variables	7	Clearly define all outcomes, exposures, predictors, potential confounders, and effect modifiers. Give diagnostic criteria, if applicable	8-9
Data sources/ measurement	8*	For each variable of interest, give sources of data and details of methods of assessment (measurement). Describe comparability of assessment methods if there is more than one group	*n/a*
Bias	9	Describe any efforts to address potential sources of bias	8
Study size	10	Explain how the study size was arrived at	7
Quantitative variables	11	Explain how quantitative variables were handled in the analyses. If applicable, describe which groupings were chosen and why	8-9
Statistical methods	12	(*a*) Describe all statistical methods, including those used to control for confounding	8-9
		(*b*) Describe any methods used to examine subgroups and interactions	8-9
		(*c*) Explain how missing data were addressed	8-9
		(*d*) *Cohort study*—If applicable, explain how loss to follow-up was addressed<br>*Case-control study*—If applicable, explain how matching of cases and controls was addressed<br>*Cross-sectional study*—If applicable, describe analytical methods taking account of sampling strategy	8-9
		(*e*) Describe any sensitivity analyses	8-9

Continued on next page

For peer review only - http://bmjopen.bmj.com/site/about/guidelines.xhtml

**Results**			
Participants	13*	(a) Report numbers of individuals at each stage of study—eg numbers potentially eligible, examined for eligibility, confirmed eligible, included in the study, completing follow-up, and analysed	n/a
		(b) Give reasons for non-participation at each stage	
		(c) Consider use of a flow diagram	
Descriptive data	14*	(a) Give characteristics of study participants (eg demographic, clinical, social) and information on exposures and potential confounders	n/a
		(b) Indicate number of participants with missing data for each variable of interest	n/a
		(c) *Cohort study*—Summarise follow-up time (eg, average and total amount)	11
Outcome data	15*	*Cohort study*—Report numbers of outcome events or summary measures over time	*n/a*
		Case-control study—Report numbers in each exposure category, or summary measures of exposure	
		Cross-sectional study—Report numbers of outcome events or summary measures	
Main results	16	(*a*) Give unadjusted estimates and, if applicable, confounder-adjusted estimates and their precision (eg, 95% confidence interval). Make clear which confounders were adjusted for and why they were included	12-15
		(*b*) Report category boundaries when continuous variables were categorized	n/a
		(*c*) If relevant, consider translating estimates of relative risk into absolute risk for a meaningful time period	n/a
Other analyses	17	Report other analyses done—eg analyses of subgroups and interactions, and sensitivity analyses	15-16
**Discussion**			
Key results	18	Summarise key results with reference to study objectives	16-18
Limitations	19	Discuss limitations of the study, taking into account sources of potential bias or imprecision. Discuss both direction and magnitude of any potential bias	19
Interpretation	20	Give a cautious overall interpretation of results considering objectives, limitations, multiplicity of analyses, results from similar studies, and other relevant evidence	20
Generalisability	21	Discuss the generalisability (external validity) of the study results	19-20
**Other information**			
Funding	22	Give the source of funding and the role of the funders for the present study and, if applicable, for the original study on which the present article is based	20

*Give information separately for cases and controls in case-control studies and, if applicable, for exposed and unexposed groups in cohort and cross-sectional studies.

Note: An Explanation and Elaboration article discusses each checklist item and gives methodological background and published examples of transparent reporting. The STROBE checklist is best used in conjunction with this article (freely available on the Web sites of PLoS Medicine at http://www.plosmedicine.org/, Annals of Internal Medicine at http://www.annals.org/, and Epidemiology at http://www.epidem.com/). Information on the STROBE Initiative is available at www.strobe-statement.org.

For peer review only - http://bmjopen.bmj.com/site/about/guidelines.xhtml
